# Design of Airport Obstacle-Free Zone Monitoring UAV System Based on Computer Vision

**DOI:** 10.3390/s20092475

**Published:** 2020-04-27

**Authors:** Liang Wang, Jianliang Ai, Li Zhang, Zhenlin Xing

**Affiliations:** Department of Aeronautics and Astronautics, Fudan University, Shanghai 200433, China; aijl@fudan.edu.cn (J.A.); 18210290016@fudan.edu.cn (L.Z.); 18110290005@fudan.edu.cn (Z.X.)

**Keywords:** quadcopter, computer vision, image processing, target identification, trajectory tracking

## Abstract

In recent years, a rising number of incidents between Unmanned Aerial Vehicles (UAVs) and planes have been reported at airports and airfields. A design scheme for an airport obstacle-free zone monitoring UAV system based on computer vision is proposed. The system integrates the functions of identification, tracking, and expelling and is mainly used for low-cost control of balloon airborne objects and small aircrafts. First, a quadcopter dynamic model and 2-Degrees of Freedom (2-DOF) Pan/Tilt/Zoom (PTZ) model are analyzed, and an attitude back-stepping controller based on disturbance compensation is designed. Second, a low and slow small-target self-identification and tracking technology is constructed against a complex environment. Based on the You Only Look Once (YOLO) and Kernel Correlation Filter (KCF) algorithms, an autonomous target recognition and high-speed tracking plan with great robustness and high reliability is designed. Third, a PTZ controller and automatic aiming strategy based on Anti-Windup Proportional Integral Derivative (PID) algorithm is designed, and a simplified, automatic-aiming expelling device, the environmentally friendly gel ball blaster, which features high speed and high accuracy, is built. The feasibility and stability of the project can be verified through prototype experiments.

## 1. Introduction

The control of air-floating objects and small low–slow aircrafts means monitoring and expelling targeted air-floating object and small low–slow aircraft through technical methods and devices. With the rapid growth of the consumer-grade Unmanned Aerial Vehicle (UAV) market, problems of illegal flying bring about risks to security and privacy. Between 19 and 21 December 2018, hundreds of flights were cancelled at Gatwick Airport near London, UK, following reports of drone sightings close to the runway. The reports caused major disruption, affecting approximately 140,000 passengers and 1000 flights [1]. Accidents caused by drones are more and more frequent, so it is necessary to regulate the use of drones. It is of great significance to deploy anti-UAV and anti-aircraft drift target systems in the airport obstacle-free zone. However, UAV reconnaissance is a challenging task due to their small size and low flying speed. Many technologies such as radar monitoring, audio monitoring, video monitoring, and Radio Frequency (RF) monitoring have the potential to detect and locate drones. Each technique has its advantages and disadvantages.

1. Radar: Radar is mainly used for the measurement and tracking of large aircraft, whereas UAVs usually fly at low altitude and low speed. Aleksander Nowak et al. introduced a method of fast, simultaneous calibration using many mobile Frequency Modulated Continuous Wave (FMCW) radars operating in the network; this is used in the anti-drone scheme [2]. Multerer et al. presented an anti-drone system that consists of a three-dimensional (3D) FMCW Multiple Input Multiple Output (MIMO) radar and a directional jammer working at 2.4 GHz [3]. Because the radar reflection section of the drone is small and the clutter reflected at low altitude is higher, the monitoring effect of drone is limited.
2. Audio: During the flight of the UAV, the motors and propellers will make sound, and the UAV will be detected, classified, and located by a system equipped with acoustic sensors. Lee et al. estimated the Direction of Arrival (DOA) of the drone by using the acoustic signal generated when the drone is flying [4]. In the vicinity of the airport, noise from aircraft engines and vehicles is very loud, so it is difficult to accurately distinguish the audio of UAV from the high ambient noise.
3. Video: Video-based detection is essentially a target detection problem in the field of computer vision and pattern recognition. Objects can be detected according to their appearance features, that is, color, contour, geometric shape, or edge [5], and their motion characteristics across successive frames [6]. You Only Look Once (YOLO) is a convolutional neural network that can predict multiple box locations and categories at one time, and can realize end-to-end target detection and recognition. Its greatest advantage is its speed, which makes it suitable for applications in the field of flight target monitoring. Ju et al. proposed a multiscale target detection approach based on YOLO V3 [7]. The YOLO algorithm is also widely used in pedestrian [8], vehicle [9], fire [10], and obstacle [11] identification, and in sports [12], medicine [13] and other monitoring fields. For airport obstacle-free zone monitoring, the combination of a flight platform, YOLO, and a target expelling device is very promising, and can effectively control and expel illegal flying targets. On the other hand, although target detection and tracking based on video images has been well studied, there are still many problems, such as the running rate, which is also a problem in target detection, tracking, and expelling.
4. RF: RF interference blocking mainly applies signal interference, acoustic interference, and other technologies to force the target aircraft to lose control of the signal. The method is only applicable to the controlled target aircraft, not to the automatic aircraft, and floating objects in the air can cause great collateral damage. When the RF signal is interrupted, the jammer is usually required to guess at the band on which the signal is running. The typical range from 2.4 to 5.0 MHz means that any technology operating within this range would also be disrupted. In an airport environment, this would be inconvenient and potentially catastrophic. It could cause unintended Electromagnetic Interference (EMI) and affect the performance of air navigation services equipment on the ground and/or onboard aircraft equipment.

Table 1 summarizes the above monitoring techniques. The detection range of the target in the table is obtained from the literature and may vary with the type of target, monitoring environment, hardware parameters, and corresponding algorithm.

Motivated by the above observations, UAV detection and expelling systems should be developed so they do not adversely impact or interfere with safe airport operations, air traffic control, and other air navigation services, or the safe and efficient operation of the National Airspace System (NAS).

The quadcopter UAV features flexible maneuverability and vertical take-off and landing, and is widely used in various fields, making it an excellent air flight platform. However, due to the restrictions of its structure and weight, the limited airborne data processing capacity, and the variety of weather conditions that can be encountered in flight, the design of the integrated identification, tracking, and expelling quadcopter UAV system based on computer vision faces a lot of challenges. Modeling and control, motion planning, perception, and mechanism design are crucial for aerial manipulations [14,15,16]. There are some challenges for UAVs when performing autonomous vision-based integrating identification, tracking, and expelling. These problems mainly come from the following aspects: (1) Due to the influence of the UAV structure strength, power configuration, flight altitude, ambient temperature and other factors, the control system design is complicated; (2) the airborne minicomputer has a limited data processing capacity; (3) the low-cost single-chip microcomputer is used to realize high-rate visual control and trigger mechanism control; (4) The flight environment of the UAV is complicated and there are many factors that restrict the efficiency. Motivated by these problems, we have systematically studied one UAV’s integrated identification, tracking, and expelling functions based on computer vision.

The contributions of this paper are as follows:

1. It has analyzed the influence of parameters such as the quadcopter frame construction, motor, and electronic speed controller, and built a quadcopter dynamic model. Considering the uncertainties of the system model, the external disturbance, and the load variation of the quadrotors, this paper designed a back-stepping attitude controller based on the disturbance compensation. It has analyzed the performance of steering gear parameters and built a 2-Degrees of Freedom (2-DOF) Pan/Tilt/Zoom (PTZ) model.
2. The low-cost single chip microcomputer Arduino (Scarmagno, Italy) was used to communicate with the computer vision module and the underlying flight control system through the serial port was used to realize high-rate visual control and PTZ control.
3. It has proposed an image processing solution based on the airborne low-cost Logitech camera (Romanel, Lausanne, Switzerland), computer Jetson nano (Santa Clara, CA, USA), and based on the YOLO algorithm, Kernel Correlation Filter (KCF) algorithm, OpenCV (Santa Clara, CA, USA) open-source computer vision library, Ubuntu 16.04 (Isle of Man, UK) operating system, and Python3 (Beaverton, OR, USA). This paper deployed the image processing environment to realize accurate and fast airborne image recognition, tracking, and locating. Based on the shooting PTZ controller designed by the Anti-Windup PID algorithm, high-accuracy aiming and fast trajectory correction were realized. Through 3D printing technology, the simulated simple launch device was designed for experimental verification.
4. By combining the ultrasonic sensors (Shenzhen, China), Lidar (Shanghai, China), GPS (Shenzhen, China) and the infrared distance measuring sensors, based on multisensor information fusion technology, this paper has constructed the UAV real-time obstacle avoidance solution centering on fuzzy control. In this process, after setting the input and output, this paper took corresponding measures to simplify the processing, formulate corresponding fuzzy rules, complete the relevant design of a fuzzy controller, and test the real-time obstacle avoidance function.

The rest of the paper is organized as follows. Section 2 gives the system overview and mathematical modeling. In Section 3, the perception and control are described. Experimental results are presented in Section 4. Concluding remarks and future work are discussed in Section 5.

## 2. System Overview and Mathematical Modeling

### 2.1. Overall System Architecture Design

**Figure 1:**
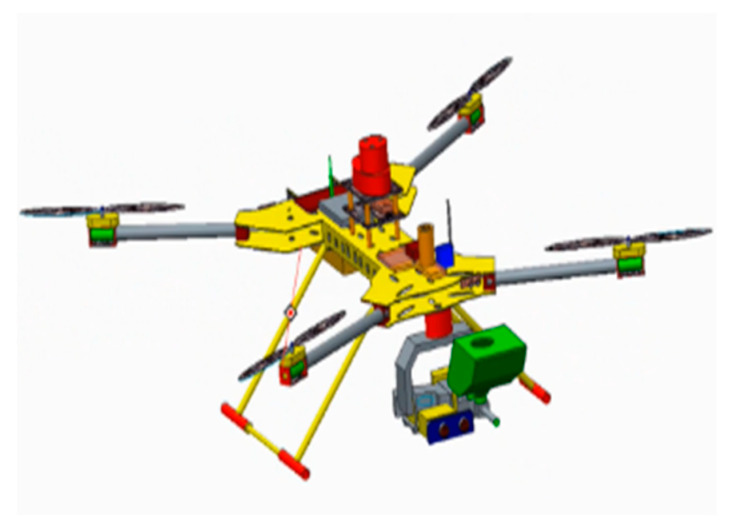
3D modeling.

**Figure 2:**
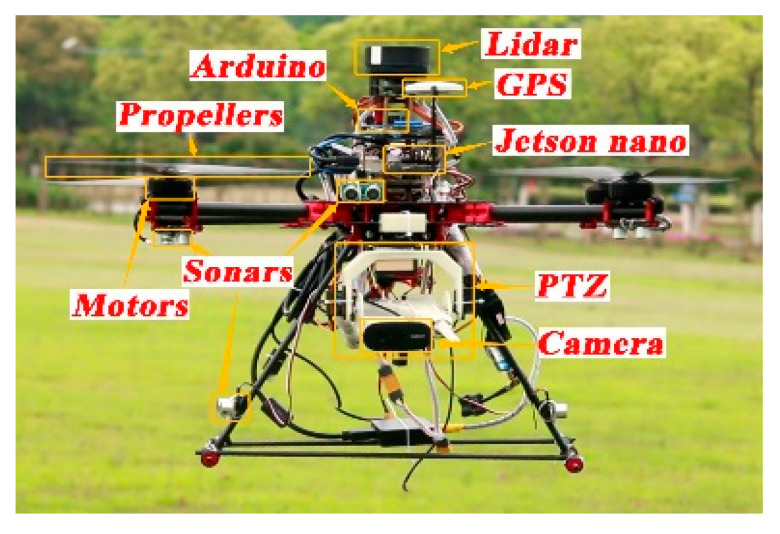
Photo of prototype.

**Figure 3:**
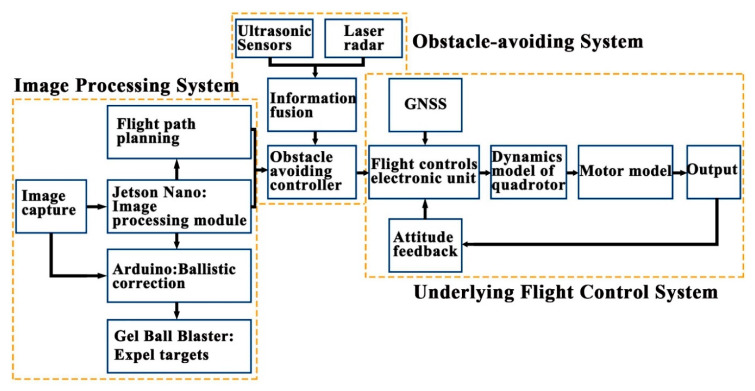
System architecture.

This paper proposes a new identification, tracking and expelling system, which can realize fully automatic, over the horizon and low-cost expulsion of a target object in flight. The design framework is divided into four parts: the underlying flight control system, the target fast recognition and tracking system, the aiming and PTZ control system, and the obstacle avoidance control system. The UAV consists of a quadcopter frame with a 680 mm wheelbase, 15 × 7 carbon fiber propellers (Shanghai, China) and 390 kV 5008 brushless motors (Shanghai, China). It is modeled with Pro/E to obtain the UAV structure model. The PIXHAWK pilot (Berkeley, CA, USA), as the bottom flight controller, runs the self-stabilization algorithm. The Jetson Nano development board is the core image processing unit, while the Arduino development board 1 is the core unit of the aiming design control system, and the Arduino development board 2, single line lidar, and ultrasonic sensor are the core units of the obstacle avoidance control system. They are shown in Figure 1, Figure 2 and Figure 3.

### 2.2. Quadcopter Dynamic Model

An accurate and reliable mathematical model is the foundation for designing a flight platform. The design and study of the flight control system have been carried out conveniently thanks to the dynamic motion simulation platform. By taking the actual flight environment into account, realistic assumptions have been proposed in this section based on the analysis of rotor aerodynamics. Equations of kinematics and dynamics that can be used for accurately describing the flight motion of a UAV (Unmanned Aerial Vehicle) have been derived from the UAV flight principle and the overall force analysis.

First of all, our assumptions are given as follows:

1. Assuming that the earth is a standard sphere, the reference ground is a standard horizontal plane and we can ignore the effect of the Earth’s rotation.
2. The quadcopter body is considered to be rigid and we can ignore its elastic deformation.
3. The air drag during the quadcopter flight is proportional to the square of the flight velocity, while the air drag direction is opposite to the velocity direction.

#### 2.2.1. Quadcopter Aerodynamic and Torque Analysis

During the quadcopter flight, forces acting on the quadcopter mainly include the lift and drag generated by the rotor, the gravity of the quadcopter, and the frictional drag generated by the quadcopter during flight.

**Figure 4:**
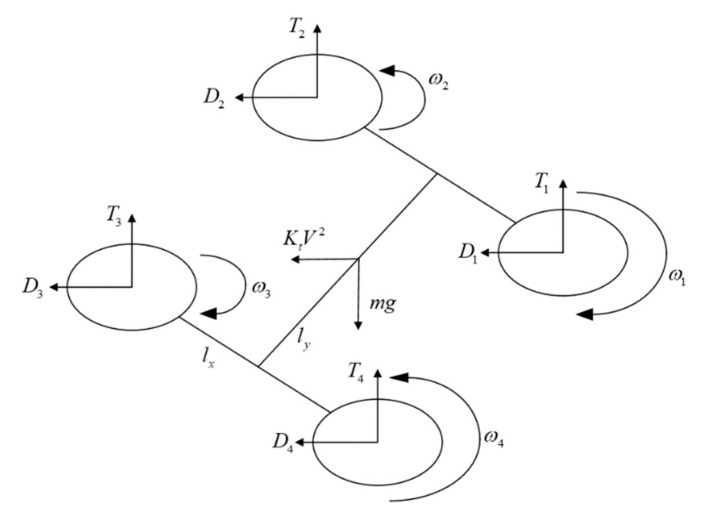
Quadcopter force analysis diagram.

The torque acting on it is primarily composed of aerodynamic torque, torque, and the rolling torque generated by the rotor, as well as the gyroscopic effect torque generated during the rotation of the rotor. The force analysis is shown in Figure 4.

Hence, the external forces received during the quadcopter flight are as follows:

(1) $$F_{SUM}=-k_{tr}{\vec{V}}^{2}+m\vec{g}+\sum_{i=1}^{4}\left({\vec{T}}_{i}+{\vec{D}}_{i}\right),$$

where $k_{tr}$ represents the frictional drag coefficient between quadcopter and air; V is its flight speed; $T_{i}$ is the lift force generated by the rotor No. i; and $D_{i}$ is the drag generated by the rotor No. i.

External torques received are presented as follows:

(2) $$\begin{array}{ll} M_{SUM} & =\left(T_{2}+T_{3}-T_{1}-T_{4}\right)l_{x}\vec{x}+\left(T_{1}+T_{2}-T_{3}-T_{4}\right)l_{y}\vec{y}\\ & +\sum_{i=1}^{4}\left(g_{ix}\vec{x}+g_{iy}\vec{y}\right)+\sum_{i=1}^{4}\left(Q_{i}\vec{z}+{\vec{L}}_{i}+{\vec{D}}_{i}h\right) \end{array},$$

where $l_{x}$ and $l_{y}$ are the horizontal distances between the rotor center and the quadcopter centroid, respectively, as shown in Figure 4; h represents the vertical distance between the rotor center and the quadcopter centroid; $g_{ix}$ and $g_{iy}$ are the gyroscopic effect stress torques generated when the rotor No. i is rotated; and $J_{r}$ is the moment of inertia of the motor and the rotor rotating group winding around the motor shaft.

When four motors are applied on the x and y axes of the body coordinate system, the total gyroscopic effect torque is presented as follows:

(3) $$Ge_{t}={\left[\begin{array}{ccc} \sum_{i=1}^{4}g_{ix} & \sum_{i=1}^{4}g_{iy} & \sum_{i=1}^{4}g_{iz} \end{array}\right]}^{T}=\left[\begin{array}{c} J_{r}q\left(\omega_{1}+\omega_{3}-\omega_{2}-\omega_{4}\right)\\ -J_{r}p\left(\omega_{1}+\omega_{3}-\omega_{2}-\omega_{4}\right)\\ 0 \end{array}\right]$$

#### 2.2.2. Definition of Coordinate System

In order to establish a state equation that can accurately describe quadcopter motion, a reasonable coordinate system should be selected with reference to specific coordinate systems for describing the relevant parameters of quadcopter linear and angular motions. This is not only a critical step in establishing the quadcopter model, but also an essential part of designing and studying the quadcopter flight control system. For better describing the quadcopter motion state, two rectangular coordinate systems are presented as follows:

1. Ground coordinate system $C_{E}$

The ground coordinate system $C_{E}:\left(O_{E},x_{E},y_{E},z_{E}\right)$ is defined. $O_{E}$ is the origin of the coordinates; the positive direction of $x_{E}$ points to the east; the positive direction of $y_{E}$ points to the south; and the positive direction of $z_{E}$ is determined to be perpendicular to the horizontal plane, pointing to the geocenter according to the right-hand rule.

2. Body coordinate system $C_{B}$

The body coordinate system is defined as $C_{B}:\left(O_{B},x_{B},y_{B},z_{B}\right)$, which is connected with the quadcopter. $O_{B}$ is the origin of coordinates, which coincides with the center of the quadcopter (all devices are included) and with $O_{E}$ at the initial moment. The $x_{B}$ axis is in the plane that passes through the centroid and is perpendicular to the connection between motors No. 1 and No. 2, pointing to the front of the body. The $y_{B}$ axis is in the plane that passes through the centroid and is perpendicular to the connection between motors No. 2 and No. 3, pointing to the right of the body. The $z_{B}$ axis is in the vertical plane passing through the centroid, pointing to the lower part of the body.

The flight motion of the quadcopter involves linear motion in three directions and angular motion winding around three axes. To be specific, the linear motion should be described in the ground coordinate system, while the angular motion should be described in the body coordinate system. In that case, the ground coordinate system and the body coordinate system should be used simultaneously in the quadcopter system modeling for describing and modeling the flight motion.

**Figure 5:**
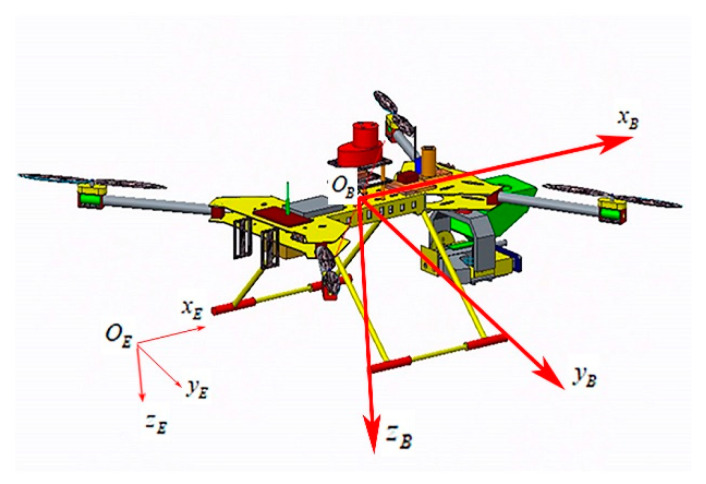
Quadcopter coordinate system.

The ground coordinate system $C_{E}$ and the body coordinate system $C_{B}$ used in the quadcopter system modeling are presented in Figure 5. It should be noted that the origins of the two coordinate systems are coincident in the initial state. Moreover, the quadcopter plane simplified structure is presented in Figure 5 to precisely demonstrate the significance of various parameters in the quadcopter model.

**Figure 6:**
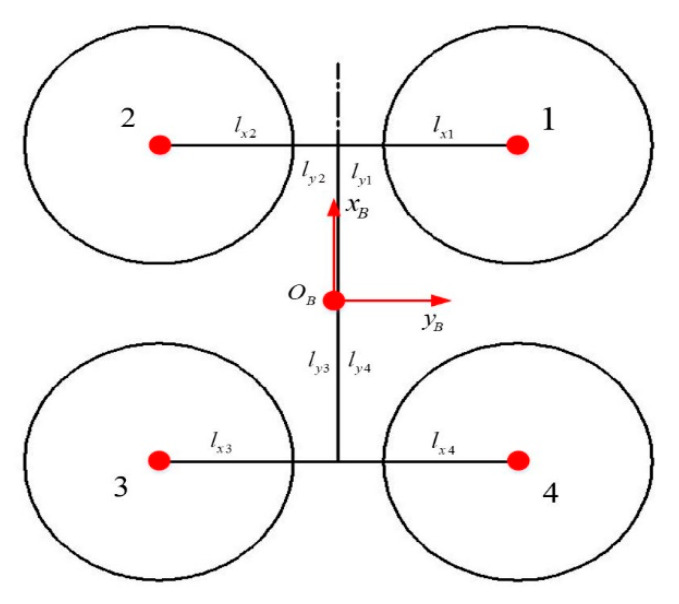
Quadcopter plane: simplified structure.

$l_{txi}$ and $l_{tyi}$ represent the distance between the motor No.i and the $x_{B}$ axis and the $y_{B}$ axis, respectively, in the quadcopter plane simplified structure shown in Figure 6. In general, the center of a multirotor quadcopter is assumed to be at the geometric center for modeling the multirotor quadcopter in the majority of the existing literature, obtaining $l_{tx1}=l_{tx2}=l_{tx3}=l_{tx4}$ and $l_{ty1}=l_{ty2}=l_{ty3}=l_{ty4}$. As a matter of fact, the center position deviates from the geometric center, affecting the control effect of the multirotor quadcopter flight control system. Therefore, considering that the center position deviates from the geometric center, the distance between each motor and the $x_{B}$ axis and the $y_{B}$ axis possesses the following relationship: $l_{tx1}=l_{tx4}$, $l_{tx2}=l_{tx3}$, $l_{ty1}=l_{ty2}$, and $l_{ty3}=l_{ty4}$.

Based on the quadcopter coordinate system shown in Figure 5, the attitude angle of quadcopter can be defined by the relationship between the body coordinate system $C_{B}$ and the ground coordinate system $C_{E}$. The attitude angle of the quadcopter in this paper can be defined as follows:

1. Roll angle ϕ: It is the angle between the body axis $O_{B}y_{B}$ and the horizontal plane, and the right roll is positive.
2. Pitch angle θ: It is the angle between the body axis $O_{B}x_{B}$ and the horizontal plane, and the up is positive.
3. Yaw angle ψ: It is the angle between the projection of the body axis $O_{B}x_{B}$ on the horizontal plane and the ground coordinate system axis $O_{E}x_{E}$, and the right yaw is positive.

#### 2.2.3. Euler Angles

The body coordinate system $C_{B}$ can be considered as the coordinate system, being fixed to the quadcopter body in the ground coordinate system $C_{E}$. $p={\left[x,y,z\right]}^{T}$ can be employed to present the position vector of the quadcopter centroid. What is more, the three attitude angles $a={\left[\phi ,\theta ,\psi \right]}^{T}$ of the quadcopter can represent the position vector of the quadcopter attitude.

The conversion matrix converting from the body coordinate system to the ground coordinate system is:

(4) $$R_{BE}=R_{\psi }R_{\theta }R_{\phi }=\left[\begin{array}{ccc} \cos\theta \cos\psi & \sin\phi \sin\theta \cos\psi -\cos\phi \sin\psi & \cos\phi \sin\theta \cos\psi +\sin\phi \sin\psi \\ \cos\theta \sin\psi & \sin\phi \sin\theta \sin\psi +\cos\phi \cos\psi & \cos\phi \sin\theta \sin\psi -\sin\phi \cos\psi \\ -\sin\theta & \sin\phi \cos\theta & \cos\phi \cos\theta \end{array}\right]$$

#### 2.2.4. Kinetic Equations

The multirotor quadcopter flight motion in the air can be decomposed into the quadcopter translation relative to the ground coordinate system and the rotation relative to the body coordinate system.

The translational motion equation of quadcopter is presented as follows:

(5) $$\left\{ \begin{array}{l} {\dot{p}}_{t}=\upsilon_{t}\\ {\dot{\upsilon }}_{t}=g{\vec{z}}_{E}-\frac{1}{m_{t}}R_{BE}F \end{array}\right.$$

where $p_{t}={\left[x_{t},y_{t},z_{t}\right]}^{T}$ and $v_{t}={\left[u_{t},v_{t},w_{t}\right]}^{T}$ represent the position and velocity of the quadcopter in the ground coordinate system, respectively; $m_{t}$ is the quadcopter mass; g is the acceleration of gravity; ${\vec{z}}_{E}={\left[0,0,1\right]}^{T}$ is the unit vector in the ground coordinate system; $R_{BE}$ is the conversion matrix from the body coordinate system to the ground coordinate system; and F is the combined external forces acting on quadcopter, except for gravity.

The attitude motion equation of quadcopter is presented as follows:

(6) $$\left\{ \begin{array}{l} {\dot{a}}_{t}=\Phi_{t}\left(a_{t}\right)\omega_{t}\\ J_{t}{\dot{\omega }}_{t}=-\left(\omega_{t}\times J_{t}\omega_{t}\right)+Ge_{t}+M \end{array}\right.$$

where $a_{t}={\left[\phi_{t},\theta_{t},\psi_{t}\right]}^{T}$ represents the attitude angle of quadcopter in the ground coordinate system; $\Phi_{t}\left(a_{t}\right)$ is the conversion matrix between the angular velocity in the body coordinate system and the Euler angular velocity in the ground coordinate system; $\omega_{t}={\left[p_{t},q_{t},r_{t}\right]}^{T}$ is the component of the rotation angular velocity of the body coordinate system on the axis of the body coordinate system relative to the ground coordinate system; $J_{t}$ is the quadcopter rotational inertia matrix; $Ge_{t}$ is the rotor-gyroscopic effect stress torque, as shown in Equation (3); and M is the combined external torque acting on the quadcopter, except for the gyroscopic effect stress torque.

By combining Equations (5) and (6), the six-degree-of-freedom nonlinear mathematical model of quadcopter can be obtained:

(7) $$\left\{ \begin{array}{l} {\dot{p}}_{t}=\upsilon_{t},{\dot{\upsilon }}_{t}=g{\vec{z}}_{E}-\frac{1}{m_{t}}R_{BE}F\\ {\dot{a}}_{t}=\Phi_{t}\left(a_{t}\right)\omega_{t},J_{t}{\dot{\omega }}_{t}=-\left(\omega_{t}\times J_{t}\omega_{t}\right)+Ge_{t}+M \end{array}\right.$$

### 2.3. PTZ Modeling

#### 2.3.1. Target Expulsion Device

**Figure 7:**
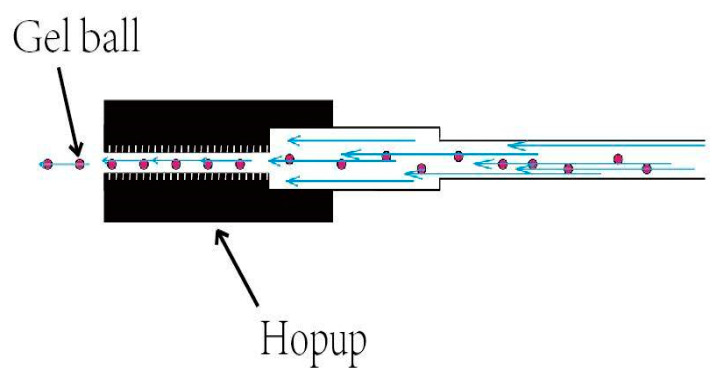
Gel ball blaster.

**Figure 8:**
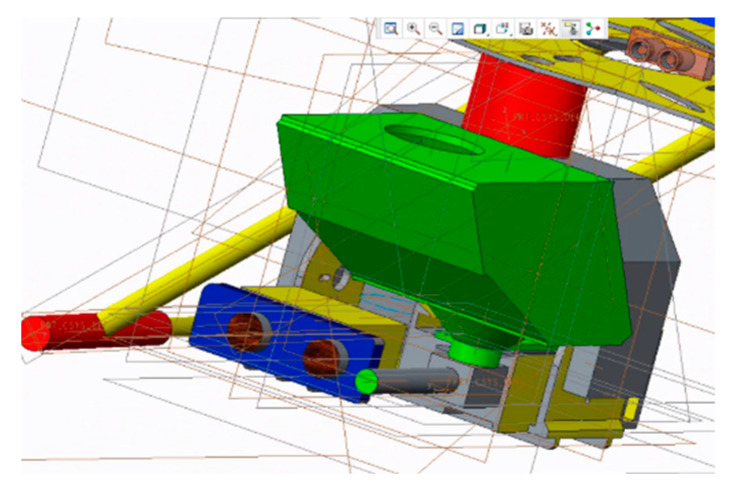
Assembly drawing of continuous gel balls feeding device.

**Figure 9:**
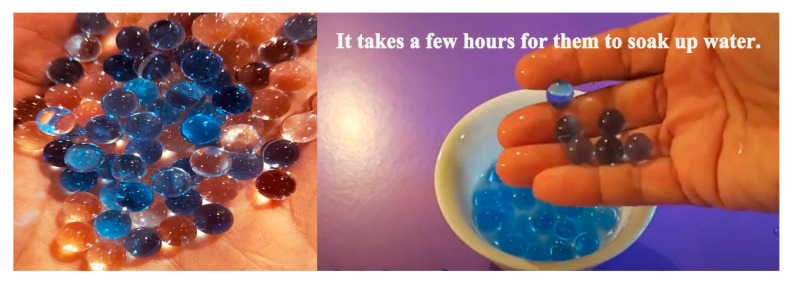
Environmentally friendly gel balls [17].

The ejection device of the launch system adopts the idea of a gel ball blaster, and is manufactured by 3D printing, with the features shown in Figure 7 and Figure 8. It is composed of a high-speed motor, a spring, a piston, a cylinder, a gear set, and a power supply. The launcher uses safe and environmentally friendly gel balls to expel targets, as shown in Figure 9.

#### 2.3.2. Design of High-Speed Steering Gear PTZ

**Figure 10:**
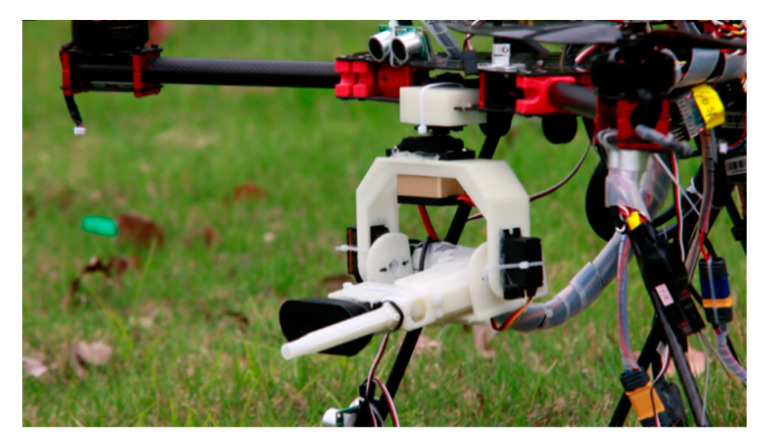
PTZ launcher.

Due to the limitations of the load and power consumption of a quadcopter, the requirements for the PTZ are being light in weight, small in volume, and simple in structure. Based on the above principles, a two-degrees-of-freedom PTZ controlled by the steering gear is designed, as shown in Figure 10, including yaw axis steering gear, a yaw axis adapter plate, pitch axis steering gear, a pitch axis adapter plate, a lens mounting base and lens, and a launcher mounting base. The model of the steering gear is ds8611, while its torque is 7.4 V 18 kg/cm and speed is 0.14 s/60°. The bracket is made using 3D printing. In order to ensure the continuous supply of gel balls for the launching device, a continuous gel ball feeding device is designed, as shown in the Figure 8. The gel balls slide downward under the action of gravity and enter the feeding hole. The designed projectile supply device can store up to 200 gel balls at a time, which can meet the requirements of expelling targets on the flight mission (see Figure 10).

The actuator design is the model obtained by identifying the test results. The operating characteristics of the steering gear include the dead band, linear zone, and nonlinear zone. The steering gear working in the linear zone can be approximately represented as a second-order model as follows:

(8) $$G(s)=\omega_{n}^{2}/\left(s^{2}+2\xi \omega_{n}s+\omega_{n}^{2}\right)$$

Since the calculation is discrete, the above equation should be discretized as follows:

(9) $$G(z)=\frac{y(z)}{u(z)}=\frac{b_{1}z^{-1}+b_{2}z^{-2}}{1-a_{1}z^{-1}-a_{2}z^{-2}}$$

where u(z) is the control signal of the actuator; y(z) is the feedback signal of the actuator; and $a_{1}$, $a_{2}$, $b_{1}$, and $b_{2}$ are the parameters obtained through identification. Actuator identification is performed using the adaptive genetic algorithm [18], obtaining $a_{1}$ = 0.7349, $a_{2}$ = −0.0076, $b_{1}$ = −0.0986, and $b_{2}$ = −0.1787.

### 2.4. Ground Station

**Figure 11:**
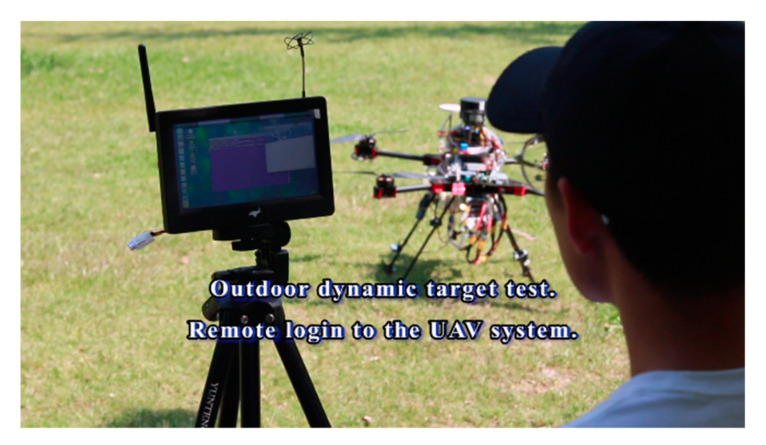
Ground station.

The ground monitoring computer is the basis of the reliable operation for the identification, tracking, and expelling system. With the help of the ground system, the remote control and monitoring of the equipment are realized. The key functions are as follows: (1) Planning the flight route of the UAV and (2) monitoring the status of the UAV in real time, while also sending instructions, as shown in Figure 11.

## 3. Perception and Control

### 3.1. Design of Backstepping Quadcopter Controller Based on Interference Compensation

The attitude controller is the most important part of multi-rotor UAV flight control system, because the performance of the attitude controller directly affects the performance of trajectory tracking. Therefore, an attitude controller with superior performance is a prerequisite for stable flight of a multi-rotor UAV flight controller. At the same time, due to the complex structure of multi-rotor UAV, it is difficult to build a precise six-freedom nonlinear mathematical model, and the flight performance of multi-rotor UAV would be affected by the uncertainties of kinetic model, external interference and other common factors. Control methods that are frequently applied nowadays include PID control [19], sliding model control [20], backstepping control [21], feedback linearization control [22], and robust control [23]. The backstepping method is especially suitable for some under-actuated systems. It converts problems related to the design of complicated and high-order controlled system into problems of low-order system decomposed from it. The method designs a virtual and intermediate control variable for each decomposed subsystem to stabilize them, and to thereby obtain the actual control input of the system through iteration. The backstepping method combines the selection of the Lyapunov function well with the solving of the control law, which proves Lyapunov liability. When designing the quadcopter controller, this thesis had fully considered the uncertainties of the kinetic model as well as the influence of external interference, and used radial basis function neural network (RBFNN) to approximate the uncertain items of the model. As for the inference brought by changes of the mass of gel balls after launching from the quadcopter as well as changes of external air current, we had used nonlinear observer to make compensation, and designed the backstepping controller based on interference compensation. Stability of the system was proved with the analysis method in Lyapunov stability theory. At the end, verification was conducted on the simulation platform, and flight test was conducted.

#### 3.1.1. Quadcopter Modeling and Processing

The nonlinear model of 6-DOF quadcopters is expressed by Equation (10):

(10) $$\left\{ \begin{array}{l} \overset{\cdot }{p_{t}}=\upsilon_{t}\\ \overset{\cdot }{a_{t}}=\Phi \left(t\right)\omega_{t}\\ \overset{\cdot }{\upsilon_{t}}=g\left[\begin{array}{c} 0\\ 0\\ 1 \end{array}\right]-\frac{1}{m_{t}}R_{BE}\left[\begin{array}{c} 0\\ 0\\ u_{t1} \end{array}\right]-\frac{k_{tr}}{m_{t}}\left[\begin{array}{c} u_{t}^{2}\\ v_{t}^{2}\\ w_{t}^{2} \end{array}\right]\\ J_{t}\overset{\cdot }{\omega_{t}}=-\left(\omega_{t}\times J_{t}\omega_{t}\right)+Ge_{t}+M_{t} \end{array}\right.$$

in which $p_{t}={\left[x_{t},y_{t},z_{t}\right]}^{T}$ is the position state vector of quadcopter in the ground coordinate system; $\upsilon_{t}={\left[u_{t},v_{t},w_{t}\right]}^{T}$ is the speed state vector of the quadcopter in the ground coordinate system; $a_{t}={\left[\phi_{t},\theta_{t},\psi_{t}\right]}^{T}$ is the attitude state vector of the quadcopter in the ground coordinate system; $\omega_{t}={\left[p_{t},q_{t},r_{t}\right]}^{T}$ is the state vector of the attitude angular velocity vector of the quadcopter in the body coordinate system; $\Phi_{t}\left(t\right)$ is the transform matrix between attitude angular velocity and Eulerian angular velocity, as shown by Equation (11); g is the local gravitational acceleration, g=9.81 m/s^2^; $m_{t}$ is the mass of the quadcopter, $m_{t}=1.94$ kg; $R_{BE}$ is the transform matrix between the body coordinate system and the ground coordinate system, as shown by Equation (12); $k_{tr}$ is the air drag coefficient of quadcopter, which is given by Equation (13); $J_{t}$ is the moment of inertia matrix of quadcopter, $J_{t}=diag\left[\begin{array}{ccc} 2.37\times 10^{-3} & 3.51\times 10^{-3} & 5.31\times 10^{-3} \end{array}\right]$ kg·m^2^; $Ge_{t}$ is the gyroscopic moment, as expressed by Equation (14); $J_{tr}$ is the rotational inertia of motor and rotary wing around the motor shaft, $J_{tr}=4.92\times 10^{-6}$ kg·m^2^; $M_{t}={\left[u_{t1},u_{t2},u_{t3},u_{t4}\right]}^{T}$ is the resultant moment excluding the gyroscopic moment, with the control variables $u_{t1},u_{t2},u_{t3},u_{t4}$ being defined by Equation (15); and $l_{t}$ is the length of the quadcopter’s frame arm, $l_{t}=0.265$ m. As shown in Table 2.

(11) $$\Phi_{t}\left(t\right)=\left[\begin{array}{ccc} 1 & \sin\phi_{t}\tan\theta_{t} & \cos\phi_{t}\tan\theta_{t}\\ 0 & \cos\phi_{t} & -\sin\phi_{t}\\ 0 & \sin\phi_{t}\sec\theta_{t} & \cos\phi_{t}\sec\theta_{t} \end{array}\right]$$

(12) $$R_{BE}=\left[\begin{array}{ccc} \cos\theta_{t}\cos\psi_{t} & \sin\phi_{t}\sin\theta_{t}\cos\psi_{t}-\cos\phi_{t}\sin\psi_{t} & \cos\phi_{t}\sin\theta_{t}\cos\psi_{t}+\sin\phi_{t}\sin\psi_{t}\\ \cos\theta_{t}\sin\psi_{t} & sin\phi_{t}\sin\theta_{t}\sin\psi_{t}+\cos\phi_{t}\cos\psi_{t} & \cos\phi_{t}\sin\theta_{t}\sin\psi_{t}-\sin\phi_{t}\cos\psi_{t}\\ -\sin\theta_{t} & \sin\phi_{t}\cos\theta_{t} & \cos\phi_{t}\cos\theta_{t} \end{array}\right]$$

(13) $$\left\{ \begin{array}{l} k_{trx}=-9.56\times 10^{-6}\times \phi_{t}^{2}+1.26\times 10^{-3}\times \phi_{t}+2\times 10^{-3}\\ k_{try}=-1.16\times 10^{-5}\times \theta_{t}^{2}+1.37\times 10^{-3}\times \theta_{t}+1\times 10^{-4}\\ k_{trz}=0.0596 \end{array}\right.$$

(14) $$Ge_{t}=\left[\begin{array}{c} J_{tr}q_{t}\left(\omega_{t1}+\omega_{t3}-\omega_{t2}-\omega_{t4}\right)\\ -J_{tr}p_{t}\left(\omega_{t1}+\omega_{t3}-\omega_{t2}-\omega_{t4}\right)\\ 0 \end{array}\right]$$

(15) $$\left\{ \begin{array}{l} u_{t1}=k_{tl}\left(\omega_{t1}^{2}+\omega_{t2}^{2}+\omega_{t3}^{2}+\omega_{t4}^{2}\right)\\ u_{t2}=k_{tl}l_{t}\left(\omega_{t2}^{2}+\omega_{t3}^{2}-\omega_{t1}^{2}-\omega_{t4}^{2}\right)\\ u_{t3}=k_{tl}l_{t}\left(\omega_{t1}^{2}+\omega_{t2}^{2}-\omega_{t3}^{2}-\omega_{t4}^{2}\right)\\ u_{t4}=k_{tt}\left(\omega_{t1}^{2}+\omega_{t3}^{2}-\omega_{t2}^{2}-\omega_{t4}^{2}\right) \end{array}\right.$$

(16) $$\left\{ \begin{array}{l} {\dot{x}}_{t}=u_{t},{\dot{y}}_{t}=v_{t},{\dot{z}}_{t}=w_{t}\\ {\dot{\phi }}_{t}=p_{t}+q_{t}\sin\phi_{t}\tan\theta_{t}+r_{t}\cos\phi_{t}\tan\theta_{t}\\ {\dot{\theta }}_{t}=q_{t}\cos\phi_{t}-r_{t}\sin\phi_{t}\\ {\dot{\psi }}_{t}=q_{t}\sin\phi_{t}\sec\theta_{t}+r_{t}\cos\phi_{t}\sec\theta_{t}\\ {\dot{u}}_{t}=-\frac{\left(\cos\phi_{t}\sin\theta_{t}\cos\psi_{t}+\sin\phi_{t}\sin\psi_{t}\right)u_{t1}+k_{trx}u_{t}^{2}}{m_{t}}+d_{tu}\\ {\dot{v}}_{t}=-\frac{\left(\cos\phi_{t}\sin\theta_{t}\sin\psi_{t}-\sin\phi_{t}\cos\psi_{t}\right)u_{t1}+k_{try}v_{t}^{2}}{m_{t}}+d_{tv}\\ {\dot{w}}_{t}=g-\frac{\cos\phi_{t}\cos\theta_{t}u_{t1}+k_{trz}w_{t}^{2}}{m_{t}}+d_{tw}\\ {\dot{p}}_{t}=\frac{q_{t}r_{t}\left(J_{y}-J_{z}\right)+u_{t2}+J_{tr}q\left(\omega_{t1}+\omega_{t3}-\omega_{t2}-\omega_{t4}\right)}{J_{x}}+d_{tp}\\ {\dot{q}}_{t}=\frac{p_{t}r_{t}\left(J_{z}-J_{x}\right)+u_{t3}-J_{tr}p\left(\omega_{t1}+\omega_{t3}-\omega_{t2}-\omega_{t4}\right)}{J_{y}}+d_{tq}\\ {\dot{r}}_{t}=\frac{p_{t}q_{t}\left(J_{x}-J_{y}\right)+u_{t4}}{J_{z}}+d_{tr} \end{array}\right.$$

Substituting the above equations into Equation (10) and introducing the external disturbance term $d_{tu},d_{tv},\cdots ,d_{tr}$, we can obtain the nonlinear model of 6-DoF quadcopters, as shown in Equation (16).

We selected the three virtual control variables as follows:

(17) $$\left\{ \begin{array}{l} u_{tx}=\frac{\left(\cos\phi_{t}\sin\theta_{t}\cos\psi_{t}+\sin\phi_{t}\sin\psi_{t}\right)u_{t1}}{m_{t}}\\ u_{ty}=\frac{\left(\cos\phi_{t}\sin\theta_{t}\sin\psi_{t}-\sin\phi_{t}\cos\psi_{t}\right)u_{t1}}{m_{t}}\\ u_{tz}=\frac{\cos\phi_{t}\cos\theta_{t}u_{t1}}{m_{t}}-g \end{array}\right.$$

Now, its translation dynamical equation can be rewritten as:

(18) $$\left\{ \begin{array}{l} {\dot{u}}_{t}=-u_{tx}-\frac{k_{trx}u_{t}^{2}}{m_{t}}+d_{tu}\\ {\dot{v}}_{t}=-u_{ty}-\frac{k_{try}v_{t}^{2}}{m_{t}}+d_{tv}\\ {\dot{w}}_{t}=-u_{tz}-\frac{k_{trz}w_{t}^{2}}{m_{t}}+d_{tw} \end{array}\right.$$

where the three virtual control variables, $u_{tx}$, $u_{ty}$, and $u_{tz}$, and the desired yaw angle $\psi_{td}$ can be used to solve the desired control variable $u_{t1}$, desired roll angle $\phi_{td}$, and desired pitch angle $\theta_{td}$.

(19) $$\left\{ \begin{array}{l} u_{t1}=m_{t}\sqrt{u_{tx}^{2}+u_{ty}^{2}+{\left(u_{tz}+g\right)}^{2}}\\ \phi_{td}=\arcsin\left(\frac{m_{t}\left(u_{tx}\sin\psi_{td}-u_{ty}\cos\psi_{td}\right)}{u_{t1}}\right)\\ \theta_{td}=\arctan\left(\frac{u_{tx}\cos\psi_{td}+u_{ty}\sin\psi_{td}}{u_{tz}+g}\right) \end{array}\right.$$

As can be seen from the nonlinear mathematical model of 6-DoF quadcopters expressed by Equation (16), the resultant external moment acting on a quadcopter leads to changes in its attitude angular velocity and thus in the attitude angle, which ultimately causes changes in the spatial position of the quadcopter. Therefore, similar to the case with the quadcopter, the trajectory tracking controller is also designed with a double closed-loop control structure comprised of an attitude controller (inner loop) and a trajectory tracking controller (outer loop). In the design of the inner-loop controller, a RBFNN is adopted to approximate uncertainties of the model, and a nonlinear observer is adopted to compensate for the influence caused by external disturbances.

#### 3.1.2. Back-Stepping Attitude Controller Design Based on Disturbance Compensation

The design and stability analysis of RBFNN and nonlinear disturbance observer in quadcopter trajectory tracking controller, as well as the demonstration of their stability, are omitted. The estimations of external disturbance terms $D_{t\upsilon }$ Equation (20) and $D_{t\omega }$ Equation (21) in the quadcopter model can be directly given by equations. The detailed process is omitted here.

(20) $$\left\{ \begin{array}{l} {\hat{D}}_{t\upsilon }\left(t\right)=L_{t\upsilon }\left(\upsilon_{t}-z_{t\upsilon }\right)\\ {\dot{z}}_{t\upsilon }=-u_{t}-\frac{k_{pr}\upsilon_{t}^{2}}{m_{t}}+{\hat{D}}_{t\upsilon }\left(t\right) \end{array}\right.$$

(21) $$\left\{ \begin{array}{l} {\hat{D}}_{t\omega }\left(t\right)=L_{t\omega }\left(\omega_{t}-z_{t\omega }\right)\\ {\dot{z}}_{t\omega }=-\left(\omega_{t}\times J_{t}\omega_{t}\right)+Ge_{t}+M_{t}+{\hat{D}}_{t\omega }\left(t\right) \end{array}\right.$$

where $u_{t}={\left[u_{tx},u_{ty},u_{tz}\right]}^{T}$, $L_{t\upsilon }>0$ and $L_{t\omega }>0$ are all parameters to be designed for the observer.

**Figure 12:**
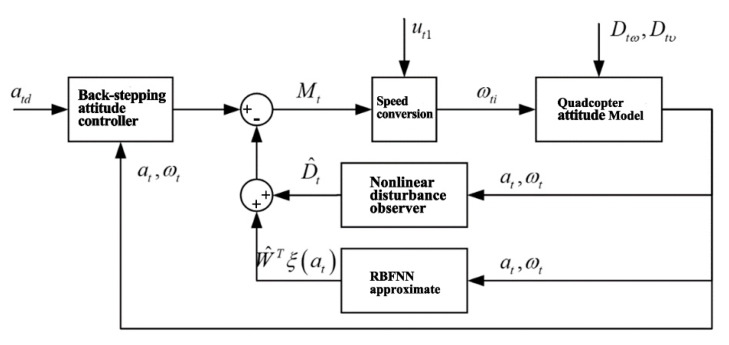
Structural diagram of quadcopter attitude controller.

Similarly, the quadcopter attitude controller is also designed with a double closed-loop structure that is comprised of an attitude angular velocity controller (inner loop) and an attitude angle controller (outer loop). The structural diagram of the attitude controller is shown in Figure 12.

Next, the process by which the attitude angle controller is designed using the back-stepping method is described.

The error between the attitude angle of the quadcopter $Y_{t}$ and the desired attitude angle $Y_{d}$ is defined as:

(22) $$e_{Y}=Y_{t}-Y_{d}$$

where $Y_{t}={\left[\phi_{t},\theta_{t},\psi_{t}\right]}^{T}$ is the attitude angle of the quadcopter and $Y_{d}={\left[\phi_{d},\theta_{d},\psi_{d}\right]}^{T}$ is the desired attitude angle.

Select a Lyapunov function for the quadcopter attitude angle system, as follows:

(23) $$V_{Y}=\frac{1}{2}e_{Y}^{T}e_{Y}$$

Taking the derivative of the function, and substituting into Equation (22), we obtain:

(24) $${\dot{V}}_{Y}=e_{Y}^{T}{\dot{e}}_{Y}=e_{Y}^{T}\left({\dot{Y}}_{t}-{\dot{Y}}_{d}\right)=e_{Y}^{T}\left(\Phi_{t}\left(t\right)X_{t}-{\dot{Y}}_{d}\right)$$

Let the command for attitude angular velocity be:

(25) $$X_{c}=-\Phi_{t}^{-1}\left(t\right)\left(\Lambda_{Y}e_{Y}-{\dot{Y}}_{d}\right)$$

where $\Lambda_{Y}=\Lambda_{Y}^{T}>0$ is the matrix to be designed.

Substituting Equation (25) into Equation (24), we obtain:

(26) $${\dot{V}}_{Y}=-e_{Y}^{T}\Lambda_{Y}e_{Y}\leq 0$$

That is, when adopting the command for attitude angular velocity designated by Equation (25), the attitude angle system of the quadcopter is stabilized.

The error between the attitude angular velocity of the quadcopter $X_{t}$ and the desired attitude angular velocity $X_{d}$ is defined as:

(27) $$e_{X}=X_{t}-X_{d}$$

where $X_{t}={\left[p_{t},q_{t},r_{t}\right]}^{T}$ is the attitude angular velocity of the quadcopter and $X_{d}={\left[p_{d},q_{d},r_{d}\right]}^{T}$ is the desired attitude angular velocity.

Taking the derivative of the above function, we can substitute it into the quadcopter attitude model as follows:

(28) $$\left\{ \begin{array}{l} {\dot{Y}}_{t}=\Phi_{t}\left(t\right)X_{t}\\ {\dot{X}}_{t}=F\left(Y_{t}\right)+G\left(Y_{t}\right)M_{t}\left(t\right)+L^{-1}W^{*T}\xi \left(Y_{t}\right)+D_{t}\left(t\right) \end{array}\right.$$

in which, $L=L^{T}>0$ is the parameter of the auxiliary system to be designed; ${\hat{D}}_{t}\left(t\right)$ represents the estimation of external unknown interference D; ${\hat{W}}^{T}\xi \left(Y_{t}\right)$ represents the RBFNN estimation of the function. Among which, $Y_{t}={\left[\phi_{t},\theta_{t},\psi_{t}\right]}^{T}$, $X_{t}={\left[p_{t},q_{t},r_{t}\right]}^{T}$, $\Phi_{t}\left(t\right)$ is as shown in Equation (1); $d_{a}\left(t\right)={\left[d_{pp},d_{pq},d_{pr}\right]}^{T}$ represents unknown external interference.

We obtain:

(29) $${\dot{e}}_{X}={\dot{X}}_{t}-{\dot{X}}_{d}=F\left(Y_{t}\right)+G\left(Y_{t}\right)M_{t}\left(t\right)+L^{-1}W^{*T}\xi \left(Y_{t}\right)+D_{t}\left(t\right)-{\dot{X}}_{d}$$

Letting

(30) $$M_{t}\left(t\right)=J_{t}\left({\dot{X}}_{d}-F\left(Y_{t}\right)-L^{-1}{\hat{W}}^{T}\xi \left(Y_{t}\right)-{\hat{D}}_{t}\left(t\right)-\Lambda_{X}e_{X}\right)$$

we have:

(31) $$\begin{array}{l} e_{X}^{T}{\dot{e}}_{X}=-e_{X}^{T}\Lambda_{X}e_{X}+e_{X}^{T}L^{-1}{\tilde{W}}^{T}\xi \left(Y_{t}\right)+e_{X}^{T}{\tilde{D}}_{t}\left(t\right)\\ \leq -e_{X}^{T}\left(\Lambda_{X}-0.5I_{3\times 3}\right)e_{X}+e_{X}^{T}L^{-1}{\tilde{W}}^{T}\xi \left(Y_{t}\right)+0.5{\left\|{\tilde{D}}_{t}\left(t\right)\right\|}^{2} \end{array}$$

Select a Lyapunov function for the quadcopter attitude angular velocity system, as follows:

(32) $$V_{X}=\frac{1}{2}e_{X}^{T}e_{X}+\frac{1}{2}e_{x}^{T}e_{x}+\frac{1}{2}tr\left({\tilde{W}}^{T}\Lambda_{W}\tilde{W}\right)+\frac{1}{2}{\tilde{D}}_{t}^{T}\left(t\right){\tilde{D}}_{t}\left(t\right)$$

Taking the derivative of the above function, and substituting it into Equation (31), we obtain:

(33) $$\begin{array}{l} {\dot{V}}_{X}=e_{X}^{T}{\dot{e}}_{X}+e_{x}^{T}{\dot{e}}_{x}+tr\left({\tilde{W}}^{T}\Lambda_{W}\dot{\tilde{W}}\right)+{\tilde{D}}_{t}^{T}\left(t\right){\dot{\tilde{D}}}_{t}\left(t\right)\\ \leq -e_{X}^{T}\left(\Lambda_{X}-0.5I_{3\times 3}\right)e_{X}+e_{X}^{T}L^{-1}{\tilde{W}}^{T}\xi \left(Y_{t}\right)+{\left\|{\tilde{D}}_{t}\left(t\right)\right\|}^{2}\\ -e_{x}^{T}\left(\Gamma -0.5I_{3\times 3}\right)e_{x}+e_{x}^{T}L^{-1}{\tilde{W}}^{T}\xi \left(Y_{t}\right)+0.5\rho^{2}+\frac{1}{2\mu }{\left\|\tilde{W}\right\|}^{2}\\ -{\tilde{D}}_{t}^{T}\left(t\right)\left(L-\left(0.5+0.5\mu \tau^{2}\right)I_{3\times 3}\right){\tilde{D}}_{t}\left(t\right)+tr\left({\tilde{W}}^{T}\Lambda_{W}\dot{\tilde{W}}\right) \end{array}$$

Observe and analyze the above equation, and select the adaptive rate of $\hat{W}$, as shown below:

(34) $$\dot{\hat{W}}=\Lambda_{W}^{-1}\left(\xi \left(Y_{t}\right){\left(e_{x}+e_{X}\right)}^{T}L^{-1}-\mu_{W}\hat{W}\right)$$

where $\Lambda_{W}=\Lambda_{W}^{-1}>0$ and $\mu_{W}>0$ are both parameters to be designed.

Substituting Equation (34) into Equation (33), we obtain:

(35) $$\begin{array}{l} {\dot{V}}_{X}\leq -e_{X}^{T}\left(\Lambda_{X}-0.5I_{3\times 3}\right)e_{X}-{\tilde{D}}_{t}^{T}\left(t\right)\left(L-\left(1.5+0.5\mu \tau^{2}\right)I_{3\times 3}\right){\tilde{D}}_{t}\left(t\right)\\ -e_{x}^{T}\left(\Gamma -0.5I_{3\times 3}\right)e_{x}+0.5\rho^{2}+\frac{1}{2\mu }{\left\|\tilde{W}\right\|}^{2}-\mu_{W}tr\left({\tilde{W}}^{T}\hat{W}\right) \end{array}$$

In the meantime, the following transformation should also be taken into account:

(36) $$2tr\left({\tilde{W}}^{T}\hat{W}\right)={\left\|\tilde{W}\right\|}^{2}+{\left\|\hat{W}\right\|}^{2}-{\left\|W\right\|}^{2}\geq {\left\|\tilde{W}\right\|}^{2}-{\left\|W\right\|}^{2}$$

Substituting Equation (36) into Equation (35), we obtain:

(37) $$\begin{array}{l} {\dot{V}}_{X}\leq -e_{X}^{T}\left(\Lambda_{X}-0.5I_{3\times 3}\right)e_{X}-{\tilde{D}}_{t}^{T}\left(t\right)\left(L-\left(0.5+1.5\mu \tau^{2}\right)I_{3\times 3}\right){\tilde{D}}_{t}\left(t\right)\\ -e_{x}^{T}\left(\Gamma -0.5I_{3\times 3}\right)e_{x}+0.5\rho^{2}-\left(\frac{\mu_{W}}{2}-\frac{1}{2\mu }\right){\left\|\tilde{W}\right\|}^{2}+\frac{\mu_{W}}{2}{\left\|W\right\|}^{2}\\ \leq -ΚV_{X}+C \end{array}$$

where

(38) $$Κ=\min\left\{\begin{array}{l} \lambda_{\min}\left(\Lambda_{X}-0.5I_{3\times 3}\right),\lambda_{\min}\left(\Gamma -0.5I_{3\times 3}\right),\\ \lambda_{\min}\left(\left(\frac{\mu_{W}}{2}-\frac{1}{2\mu }\right)/\lambda_{\max}\left(\Lambda \right)\right),\lambda_{\min}\left(L-\left(0.5+1.5\mu \tau^{2}\right)I_{3\times 3}\right) \end{array}\right\}$$

(39) $$C=0.5\rho^{2}+\frac{\mu_{W}}{2}{\left\|W\right\|}^{2}$$

To ensure the stability of the attitude angular velocity system of the quadcopter, the parameters of the controller and relevant auxiliary systems should at least satisfy the following conditions:

(40) $$\begin{array}{l} \Lambda_{X}-0.5I_{3\times 3}>0,\;\Gamma -0.5I_{3\times 3}>0,\\ \frac{\mu_{W}}{2}-\frac{1}{2\mu }>0,\;L-\left(0.5+1.5\mu \tau^{2}\right)I_{3\times 3}>0. \end{array}$$

Meanwhile, as known from literature [24,25],

(41) $$0\leq V_{X}\leq \frac{C}{Κ}+\left(V_{X}\left(0\right)-\frac{C}{Κ}\right)e^{-Kt}$$

where $V_{X}$ is convergent and $\underset{t\to \infty }{\lim}V_{X}=\frac{C}{K}$.

Thus, under the effect of the control moment for attitude angle, as shown by Equation (30), the attitude angular velocity system of the quadcopter is stable.

#### 3.1.3. Trajectory Tracking Control Based on the Back-Stepping Method

As can be known from the above, the model of the quadcopter position system is as follows:

(42) $$\left\{ \begin{array}{l} {\dot{x}}_{t}=u_{t}\\ {\dot{y}}_{t}=v_{t}\\ {\dot{z}}_{t}=w_{t}\\ {\dot{u}}_{t}=-\frac{\left(\cos\phi_{t}\sin\theta_{t}\cos\psi_{t}+\sin\phi_{t}\sin\psi_{t}\right)u_{t1}+k_{trx}u_{t}^{2}}{m_{t}}+d_{tu}\\ {\dot{v}}_{t}=-\frac{\left(\cos\phi_{t}\sin\theta_{t}\sin\psi_{t}-\sin\phi_{t}\cos\psi_{t}\right)u_{t1}+k_{try}v_{t}^{2}}{m_{t}}+d_{tv}\\ {\dot{w}}_{t}=g-\frac{\cos\phi_{t}\cos\theta_{t}u_{t1}+k_{trz}w_{t}^{2}}{m_{t}}+d_{tw} \end{array}\right.$$

The quadcopter position system shown by Equation (42) is divided into three subsystems along the longitudinal and horizontal movements, which are described in the state space:

(43) $$\begin{array}{l} \left\{ \begin{array}{l} {\dot{y}}_{1}={\dot{x}}_{t}=u_{t}=x_{1}\\ {\dot{x}}_{1}={\dot{u}}_{t}=-\frac{\left(\cos\phi_{t}\sin\theta_{t}\cos\psi_{t}+\sin\phi_{t}\sin\psi_{t}\right)u_{t1}+k_{trx}u_{t}^{2}}{m_{t}}+d_{tu} \end{array}\right.\\ \left\{ \begin{array}{l} {\dot{y}}_{2}={\dot{y}}_{t}=v_{t}=x_{2}\\ {\dot{x}}_{2}={\dot{v}}_{t}=-\frac{\left(\cos\phi_{t}\sin\theta_{t}\sin\psi_{t}-\sin\phi_{t}\cos\psi_{t}\right)u_{t1}+k_{try}v_{t}^{2}}{m_{t}}+d_{tv} \end{array}\right.\\ \left\{ \begin{array}{l} {\dot{y}}_{3}={\dot{z}}_{t}=w_{t}=x_{3}\\ {\dot{x}}_{3}={\dot{w}}_{t}=g-\frac{\cos\phi_{t}\cos\theta_{t}u_{t1}+k_{trz}w_{t}^{2}}{m_{t}}+d_{tw} \end{array}\right. \end{array}$$

The following part takes the height controller as an example, where the back-stepping method is adopted in design.

The height error of the quadcopter is defined as follows:

(44) $$e_{y3}=y_{3d}-y_{3}$$

where $y_{3d}$ is the desired height of the quadcopter and $y_{3}$ is its actual height.

Select a Lyapunov function for the quadcopter height subsystem, as follows:

(45) $$V_{y3}=\frac{1}{2}e_{y3}^{2}$$

Taking the derivative of the above Lyapunov function, we obtain:

(46) $${\dot{V}}_{y3}=e_{y3}{\dot{e}}_{y3}=e_{y3}\left({\dot{y}}_{3d}-x_{3}\right)=e_{y3}\left({\dot{y}}_{3d}-w_{p}\right)$$

Design the following virtual control variable according to Equation (46):

(47) $$w_{pd}={\dot{z}}_{pd}+\Lambda_{y3}e_{y3}$$

where $\Lambda_{y3}>0$ is the controller parameter to be designed.

Substituting Equation (47) into Equation (46), we obtain:

(48) $${\dot{V}}_{y3}=e_{y3}\left({\dot{y}}_{3d}-{\dot{y}}_{3d}-\Lambda_{y3}e_{y3}\right)=-\Lambda_{y3}e_{y3}^{2}\leq 0$$

that is, the virtual control variable designed according to Equation (47) enables the error of quadcopter height to converge.

The longitudinal speed error of the quadcopter is defined as follows:

(49) $$e_{x3}=x_{3}-x_{3d}=x_{3}-{\dot{z}}_{pd}-\Lambda_{y3}e_{y3}$$

where $x_{3d}$ is the desired longitudinal speed of the quadcopter and $x_{3}$ is its actual longitudinal speed.

Taking the derivative of the longitudinal speed of the quadcopter, we obtain:

(50) $${\dot{e}}_{x3}={\dot{x}}_{3}-{\ddot{z}}_{td}-\Lambda_{y3}{\dot{e}}_{y3}=-u_{tz}-\frac{k_{trz}w_{t}^{2}}{m_{t}}+d_{tw}-{\ddot{z}}_{td}-\Lambda_{y3}{\dot{e}}_{y3}$$

Furthermore, we have:

(51) $${\dot{e}}_{y3}={\dot{y}}_{3d}-{\dot{y}}_{3}=w_{td}-w_{t}-\Lambda_{y3}e_{y3}=-e_{x3}-\Lambda_{y3}e_{y3}$$

Substituting Equation (51) into Equation (50), we obtain:

(52) $${\dot{e}}_{x3}={\dot{x}}_{3}-{\ddot{z}}_{td}-\Lambda_{y3}{\dot{e}}_{y3}=-u_{z}-\frac{k_{prz}w_{p}^{2}}{m_{p}}+d_{pw}-{\ddot{z}}_{pd}+\Lambda_{y3}\left(e_{x3}+\Lambda_{y3}e_{y3}\right)$$

where $\Lambda_{x3}>0$ is the controller parameter to be designed.

Substituting Equation (52) into Equation (51), we obtain:

(53) $${\dot{e}}_{x3}=-\Lambda_{x3}e_{x3}+e_{y3}+d_{tw}-{\hat{d}}_{tw}$$

Select a Lyapunov function for the quadcopter longitudinal speed subsystem, as follows:

(54) $$V_{x3}=\frac{1}{2}e_{y3}^{2}+\frac{1}{2}e_{x3}^{2}+\frac{1}{2}{\tilde{d}}_{tw}^{2}$$

where ${\tilde{d}}_{tw}=d_{tw}-{\hat{d}}_{tw}$ is the estimated error of the nonlinear observer.

Taking the derivative of the Lyapunov function, as shown by Equation (54), we obtain:

(55) $$\begin{array}{l} {\dot{V}}_{x3}=e_{y3}{\dot{e}}_{y3}+e_{x3}{\dot{e}}_{x3}+{\tilde{d}}_{tw}{\dot{\tilde{d}}}_{tw}\\ =e_{y3}\left(-e_{x3}-\Lambda_{y3}e_{y3}\right)+e_{x3}\left({\tilde{d}}_{tw}-\Lambda_{x3}e_{x3}+e_{y3}\right)+{\tilde{d}}_{tw}{\dot{\tilde{d}}}_{tw}\\ =-\Lambda_{y3}e_{y3}^{2}-\Lambda_{x3}e_{x3}^{2}+e_{x3}{\tilde{d}}_{tw}+{\tilde{d}}_{tw}{\dot{\tilde{d}}}_{tw}\\ \leq -\Lambda_{y3}e_{y3}^{2}-\Lambda_{x3}e_{x3}^{2}+0.5e_{x3}^{2}+0.5{\tilde{d}}_{tw}^{2}+{\tilde{d}}_{tw}{\dot{\tilde{d}}}_{tw} \end{array}$$

In the meantime, we use the nonlinear observer to estimate the external longitudinal disturbance term $d_{tw}$. The specific definition is shown below. The demonstration process of its stability is omitted here.

(56) $$\left\{ \begin{array}{l} {\hat{d}}_{tw}\left(t\right)=L_{w}\left(w_{t}-z_{w}\right)\\ {\dot{z}}_{w}=-u_{z}-\frac{k_{trz}w_{t}^{2}}{m_{t}}+{\hat{d}}_{tw}\left(t\right) \end{array}\right.$$

According to Equation (56), we can obtain:

(57) $$\begin{array}{l} {\dot{\hat{d}}}_{tw}\left(t\right)=L_{w}\left({\dot{w}}_{t}-{\dot{z}}_{w}\right)=L_{w}\left({\dot{w}}_{t}+u_{z}+\frac{k_{trz}w_{t}^{2}}{m_{t}}-{\hat{d}}_{tw}\left(t\right)\right)\\ =L_{w}\left(d_{tw}\left(t\right)-{\hat{d}}_{tw}\left(t\right)\right)=L_{w}{\tilde{d}}_{tw}\left(t\right) \end{array}$$

Substituting Equation (57) into Equation (56), we obtain:

(58) $$\begin{array}{l} {\dot{V}}_{x3}\leq -\Lambda_{y3}e_{y3}^{2}-\Lambda_{x3}e_{x3}^{2}+0.5e_{x3}^{2}+0.5{\tilde{d}}_{tw}^{2}+{\tilde{d}}_{tw}{\dot{\tilde{d}}}_{tw}\\ \leq -\Lambda_{y3}e_{y3}^{2}-\Lambda_{x3}e_{x3}^{2}+0.5e_{x3}^{2}+0.5{\tilde{d}}_{tw}^{2}+{\tilde{d}}_{tw}\left({\dot{d}}_{tw}-{\dot{\hat{d}}}_{tw}\right)\\ \leq -\Lambda_{y3}e_{y3}^{2}-\Lambda_{x3}e_{x3}^{2}+0.5e_{x3}^{2}+0.5{\tilde{d}}_{tw}^{2}+{\tilde{d}}_{tw}\left({\dot{d}}_{tw}-L_{w}{\tilde{d}}_{tw}\right)\\ \leq -\Lambda_{y3}e_{y3}^{2}-\Lambda_{x3}e_{x3}^{2}+0.5e_{x3}^{2}+0.5{\tilde{d}}_{tw}^{2}+{\tilde{d}}_{tw}{\dot{d}}_{tw}-L_{w}{\tilde{d}}_{tw}^{2}\\ \leq -\Lambda_{y3}e_{y3}^{2}-\Lambda_{x3}e_{x3}^{2}+0.5e_{x3}^{2}+0.5{\tilde{d}}_{tw}^{2}+0.5{\tilde{d}}_{tw}^{2}+0.5{\dot{d}}_{tw}^{2}-L_{w}{\tilde{d}}_{tw}^{2}\\ \leq -\Lambda_{y3}e_{y3}^{2}-\left(\Lambda_{x3}-0.5\right)e_{x3}^{2}-\left(L_{w}-0.5\right){\tilde{d}}_{tw}^{2}+0.5\mu_{dw}^{2} \end{array}$$

To ensure the convergence of the quadcopter’s longitudinal speed error, the following condition should be satisfied:

(59) $$\Lambda_{y3}>0,\;\Lambda_{x3}-0.5>0,\;L_{w}-0.5>0$$

By the same token, we can derive the virtual control variables for the two subsystems along horizontal movements, as shown below:

(60) $$\begin{array}{l} u_{td}={\dot{x}}_{td}+\Lambda_{y1}e_{y1}\\ u_{x}={\ddot{x}}_{td}+e_{y1}-\Lambda_{y1}\left(e_{x1}+\Lambda_{y1}e_{y1}\right)-\Lambda_{x1}e_{x1}+\frac{k_{trx}u_{t}^{2}}{m_{t}}-{\hat{d}}_{tu}\\ v_{td}={\dot{y}}_{td}+\Lambda_{y2}e_{y2}\\ u_{y}={\ddot{y}}_{td}+e_{y2}-\Lambda_{y2}\left(e_{x2}+\Lambda_{y2}e_{y2}\right)-\Lambda_{x2}e_{x2}+\frac{k_{try}v_{t}^{2}}{m_{t}}-{\hat{d}}_{tv} \end{array}$$

where $e_{y1}=y_{1d}-y_{1}$ and $e_{y2}=y_{2d}-y_{2}$ are horizontal position errors; $e_{x1}=x_{1}-x_{1d}$ and $e_{x2}=x_{2}-x_{2d}$ are horizontal speed errors; and $\Lambda_{x2}-0.5>0$, $\Lambda_{x1}-0.5>0$, $\Lambda_{y2}>0$ and $\Lambda_{y1}>0$ are controller parameters to be designed.

### 3.2. Real-Time Image Recognition, Location, and Tracking Based on the YOLO and KCF Algorithms

#### 3.2.1. Target Recognition, Location

The deep learning model has become a hot research topic in computer vision due to its strong presentation ability, data accumulation, and computing power progress. It is mainly divided into three levels: classification, detection, and segmentation.

The classification task focuses on the whole, giving the content description of the whole picture, while the detection focuses on the specific object target, requiring the simultaneous acquisition of the class information and location information of the target. Compared with classification, detection is the understanding of the foreground and background of a picture. We need to isolate an interesting target from the background and determine the target description (class and location). Therefore, the output of a detection model is a list, and each item in the list uses a dataset, giving the class and position of the detected target (often expressed by coordinates of a rectangular detection box).

With the development of Deep Neural Networks (DNN), the Convolutional Neural Network (CNN) has been widely used in image recognition. When a video collected by the camera is subject to frame extracting to obtain the image, the first task is to detect all kinds of aircrafts in the image, that is, target detection of the corresponding image processing task. However, CNN can only judge if the target object appears in the image, but cannot locate the position of the target object in the image. Ross et al. [26], from Berkeley University, proposed a new network structure named Regions with Convolutional Neural Network Features (R-CNN) in 2014, which realized the functions of image recognition and item location. Girshick proposed a new Faster R-CNN [27] in 2016. In structure, Faster R-CNN has already integrated feature extraction, proposal, bounding box regression, and classification in one network, greatly improving the overall performance, especially in detection speed. The improved versions of Fast R-CNN [28] and Faster R-CNN realized higher recognition accuracy, but the application of classifiers in the R-CNN series network caused the processing speed of Fast R-CNN to be 0.5 frames per second (FPS), while that of Faster R-CNN reached 7 FPS. This is a great improvement compared to R-CNN, but still fails to meet the real-time requirement.

In 2016, Redmon et al. [29,30,31] from the University of Washington proposed a real-time object detection network YOLO (You Only Look Once). The YOLO algorithm is also a classic algorithm in the field of target detection. The core idea is to use the entire picture as an input to the network, directly returning the location of the bounding box and its class in the output layer. The object detection problem is transformed into a regression problem for processing and a single neural network can be used to obtain the position coordinates and relative size of the object from one image. On a computer with a GPU, YOLO in its standard version can process an image on the real-time basis at a rate of 45 FPS, but the YOLO fast version can reach a speed of 155 FPS, doubling the average accuracy of any other real-time object detection method.

We compared the similarities and differences of Faster R-CNN and YOLOv3 in target detection. With regard to the traditional multiclass detection task, the target detection task in high-speed field is relatively simple (for aircraft detection only). In terms of accuracy, YOLOv3 has better performance; in terms of processing speed, Faster R-CNN algorithm runs more slowly: the time spent on one image is 5–6 s, which does not meet the speed requirement in actual testing tasks. However, the YOLOv3 algorithm takes only 0.1–0.2 s to process one image and satisfies the requirement better in actual conditions. To improve the accuracy of lane detection in complex scenarios, an adaptive lane feature learning algorithm that can automatically learn the features of a lane in various scenarios is proposed [32]. As a result, the YOLOv3 algorithm is adopted for target detection.

**Figure 13:**
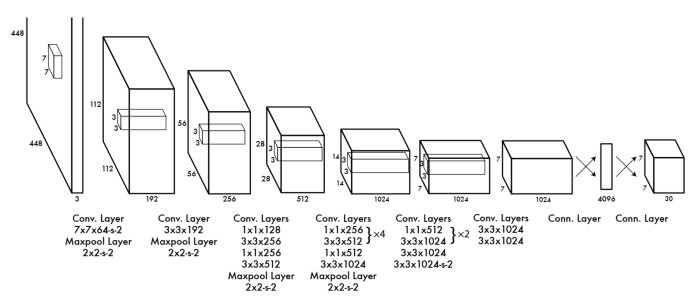
YOLO network architecture.

This paper aims to propose a real-time image recognition, location, and tracking system on the basis of a YOLO network. YOLO divides the whole input image into a S×S grid that can predict the normalized relative coordinates (x,y), normalized relative length and width (w,h), and confidence level conf of the central position of R bounding boxes, as well as C conditional probabilities, that is, the probability that this object belongs to one class when this grid contains the target object. The YOLO network structure is shown in Figure 13, including 24 convolutional layers and two fully connected layers. Convolutional layers are used to extract the features in the image, while the fully connected layers are used to build the relationship between the features and the probability of image position and target. The output of the YOLO network is a S×S×(5×R+C) tensor, where each 1×1×(5×R+C) tensor corresponds to one S×S grid from the image, including conditional probability, bounding box size, and coordinate information.

The loss function of YOLO is shown in Equation (61):

(61) $$loss=\eta_{1}+\eta_{2}+\eta_{3}+\eta_{4}$$

where $\eta_{1}$ is shown in Equation (62), representing the prediction of bounding box coordinate and its size; $\eta_{2}$ is shown in Equation (63), representing the confidence level prediction of a bounding box containing a target object; $\eta_{3}$ is shown in Equation (64), representing the confidence level prediction of a bounding box without a target object; and $\eta_{4}$ is shown in Equation (65), representing the conditional class probability prediction.

(62) $$\begin{array}{ll} \eta_{1} & =\lambda_{coord}\sum_{i=0}^{S^{2}}\sum_{j=0}^{R}1_{ij}^{obj}\left[{\left(x_{i}-{\hat{x}}_{i}\right)}^{2}+{\left(y_{i}-{\hat{y}}_{i}\right)}^{2}\right]\\ & +\lambda_{coord}\sum_{i=0}^{S^{2}}\sum_{j=0}^{R}1_{ij}^{obj}\left[{\left(\sqrt{w_{i}}-\sqrt{{\hat{w}}_{i}}\right)}^{2}+{\left(\sqrt{h_{i}}-\sqrt{{\hat{h}}_{i}}\right)}^{2}\right] \end{array}$$

(63) $$\eta_{2}=\sum_{i=0}^{S^{2}}\sum_{j=0}^{R}1_{ij}^{obj}{\left(c_{i}-{\hat{c}}_{i}\right)}^{2}$$

(64) $$\eta_{3}=\lambda_{noobj}\sum_{i=0}^{S^{2}}\sum_{j=0}^{R}1_{ij}^{noobj}{\left(c_{i}-{\hat{c}}_{i}\right)}^{2}$$

(65) $$\eta_{4}=\sum_{i=0}^{S^{2}}1_{i}^{obj}\sum_{a\in classes}{\left(p_{i}\left(a\right)-{\hat{p}}_{i}\left(a\right)\right)}^{2}$$

where $\lambda_{coord}$ is the predicted weight of the bounding box coordinate; $\lambda_{noobj}$ is the confidence weight of bounding box without the target object; $1_{ij}^{obj}$ is used to judge if the j bounding box in i grid is responsible for the object; and $1_{i}^{obj}$ is used to judge whether there is an object center in i grid.

Through this loss of function, YOLO can achieve a balance between the bounding box coordinates and size, confidence, and conditional probability. In YOLO, there will be multiple bounding boxes in one grid conducting the prediction of the object. However, in the training process, it is hoped that each object can be predicted by only one bounding box in the end. Therefore, if the IOU (Intersection Over Union) of one current bounding box prediction to the GTB (Ground True Box) is the highest, this bounding box will be in charge of the prediction of this object. As the training progresses, each bounding box will provide a better prediction of the responsible object.

Meanwhile, when YOLO is used for real-time location of an aircraft or a target, it is only needed to recognize the single object, the aircraft, so C = 1. Moreover, because the output of YOLO contains bounding box coordinate and size, it is required to transmit the coordinate and size of the bounding box in the image into the actual location and distance of the aircraft. Therefore, the last fully connected layer is added to the YOLO network to build the relationship between the coordinate and size of the bounding box and the actual location and distance of the aircraft.

**Figure 14:**
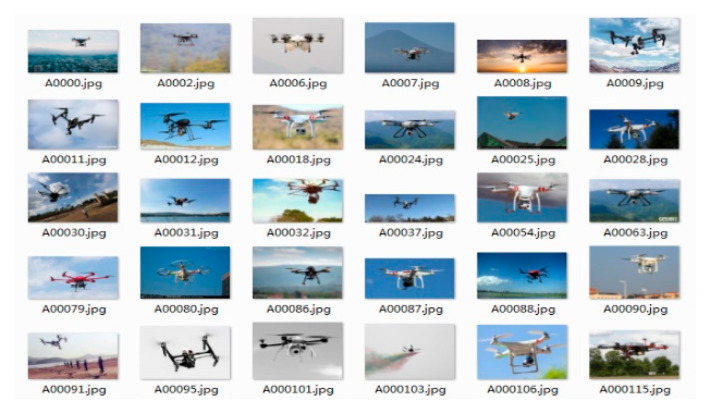
Rotorcraft positive specimen training set.

**Figure 15:**
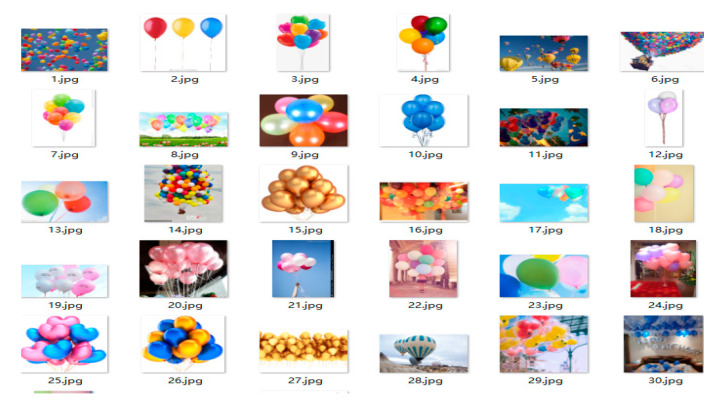
Balloon positive specimen training set.

**Figure 16:**
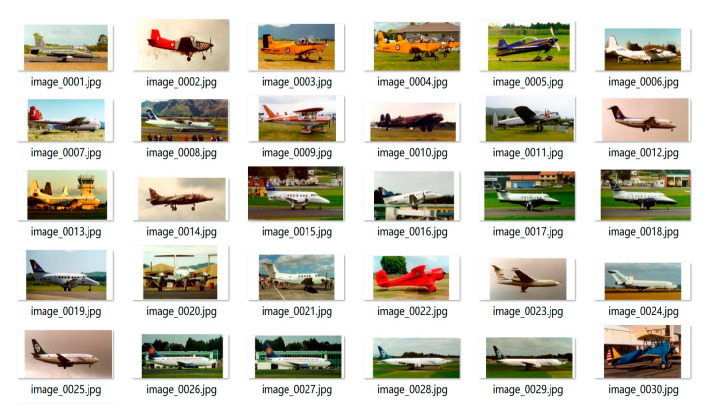
Fixed-wing aircraft positive specimen training set.

**Figure 17:**
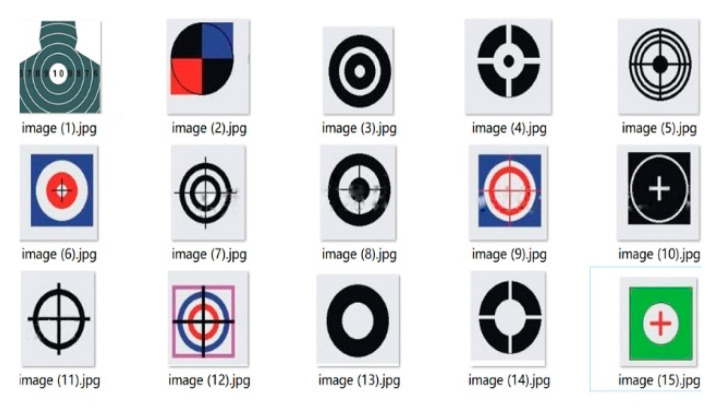
Target positive specimen training set.

**Figure 18:**
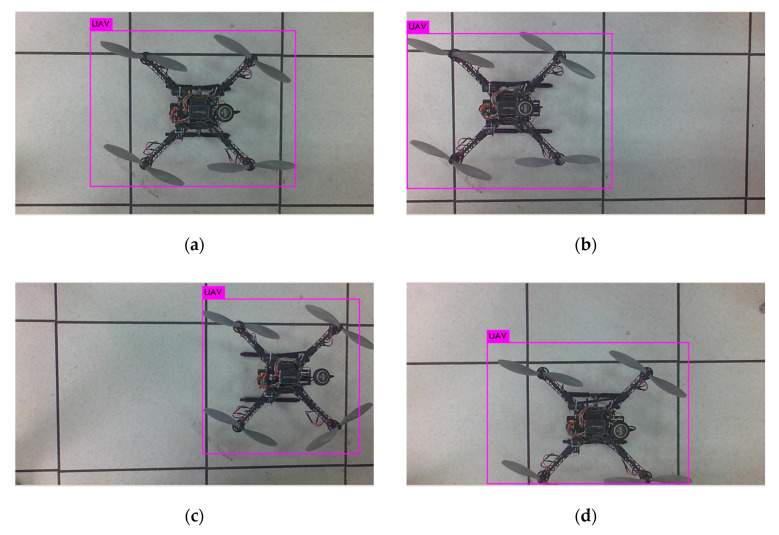
YOLO real-time recognition location UAV. (**a**–**d**) Four sets of localization experiments.

In network training, 2000 images are collected as the specimens, and the locations of the targets in sample images are marked. Some specimens are shown as Figure 14, Figure 15, Figure 16 and Figure 17. Figure 18 provides the recognition of targets by YOLO after training.

As seen in Table 3, when the UAV is away from the image boundary, it can be located accurately, but when the UAV approaches the image boundary or goes beyond the image range, the accuracy of the location may decrease.

#### 3.2.2. Target Tracking

In the early stage, image tracking algorithms such as Camshift, light stream, and background subtraction were very popular, and were applied successfully in static background conditions. After 2008, such methods were gradually abandoned, with the research focus shifting to the study of image tracking with a dynamic or complex background.

Currently, OpenCV provides eight algorithms in target tracking: Boosting, Channel and Spatial Reliability Tracking (CSRT), GOTURN, KCF, Median Flow, Multiple Instance Learning (MIL), Minimum Output Sum of Squared Error (MOSSE), and Tracking Learning Detection (TLD), including the classic algorithm and the current advanced tracking algorithm.

The main contributions of the KCF algorithm are as follows: (1) Positive and negative samples are collected by using the cyclic matrix of the surrounding area of the target, and the target detector is trained by using ridge regression. The operation of a matrix is transformed into a Hadamard product of vector by the diagonalization property of a cyclic matrix in Fourier space, i.e., the dot product of an element, greatly reducing the amount of computation, improving the speed of operation, and making the algorithm meet the real-time need. (2) The ridge regression of linear space is mapped to the nonlinear space by a kernel function. In the nonlinear space, by solving a dual problem and some common constraints, the calculation can also be simplified by diagonalizing the cyclic matrix Fourier space. (3) A way to integrate multichannel data into the algorithm is presented. The histogram of oriented gradient (HOG) feature is used when the features of a targeted area are extracted. The HOG feature divides the image into smaller parts called cells. Gradient information is extracted from the cells, and a gradient orientation histogram is drawn to reduce the influence of light. By gathering the orientation histograms of several cells for block normalization, all orientation histograms of cells are connected in a series to get the features of the image.

The accelerating methods used in KCF are as follows: (1) Detection: the cyclic matrix + Fourier transform calculation response diagram are used; the original $O(N^{3})$ algorithm needs only $O\left(n^{*}\log(n)\right)$. (2) Training: The cyclic matrix property is used for training in the frequency domain. (3) Kernel regression acceleration: For a kernel function, it can also be converted to the frequency domain for training and detection, greatly improving the speed. (4) Special kernel functions are further accelerated: for a Gaussian kernel, a polynomial kernel can further use a cyclic matrix to calculate the cyclic matrix of kernel functions.

We have built the OpenCV running environment on the Jetson nano and deployed the YOLO algorithm and KCF tracker [33]. Then we prepared for the next test.

### 3.3. Automatic Tracking Strategy and PTZ Control

#### 3.3.1. Principle of Visual Feedback Servo Tracking

Through image acquisition and comparison of successive frames, computer vision feedback can be realized. The difference between the target position extracted according to the next image information and the position information extracted from the previous image is used as the input signal of PTZ position control; the space movement of the moving target is converted into frame image plane coordinates, and the angle of rotation required for PTZ aiming is calculated to form the visual feedback.

**Figure 19:**
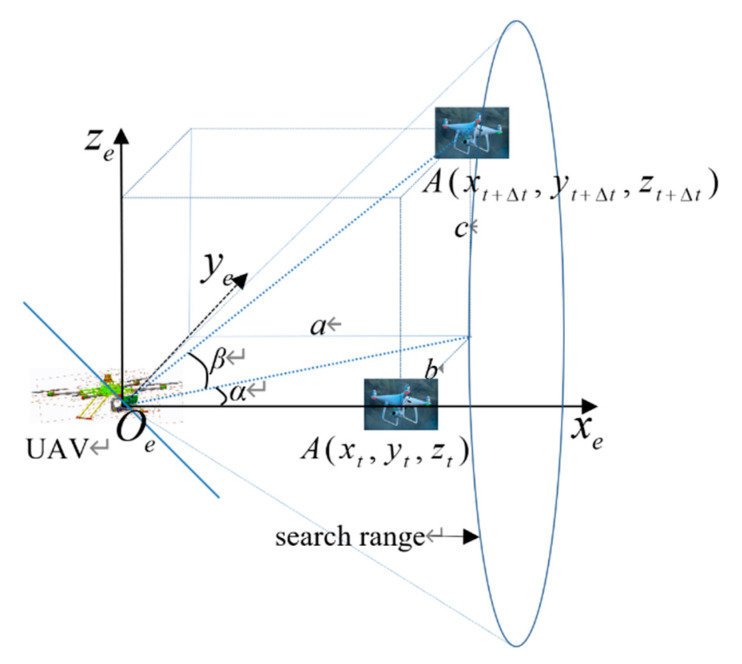
2-DOF PTZ tracking coordinate diagram.

As shown in Figure 19, the optical center point of the camera mounted on the PTZ is used as the reference point $O_{e}$, and a space reference coordinate system $O_{e}x_{e}y_{e}z_{e}$ is built. By means of the space reference coordinate system and image plane coordinate transformation, the coordinate of the target in the camera imaging plane, the image plane coordinate, is determined. Assuming that at moment t, the coordinate of the target object in the reference coordinate system $O_{e}x_{e}y_{e}z_{e}$ is $A(x_{t},y_{t},z_{t})$, after image plane coordinate transformation, its location coordinate $A(x_{t1},y_{t1},)$ in the image plane can be determined. After a very short time Δt, PTZ does not act; the space reference coordinate system remains unchanged and the coordinate of the target object in the reference coordinate system is $A(x_{t+\Delta t},y_{t+\Delta t},z_{t+\Delta t})$. After image plane coordinate transformation, its location coordinate $A(x_{t1+\Delta t},y_{t1+\Delta t},)$ in the image plane can be determined. After calculation, within the time interval Δt, the coordinate of the target object turns α and β relative to the $x_{e}$ axis and $y_{e}$ axis of the space reference coordinate system.

Assuming that the execution time of the PTZ action is 0, after the PTZ location adjustment, the space datum coordinate system becomes $O^{\prime }{}_{e+\Delta t}x^{\prime }{}_{e+\Delta t}y^{\prime }{}_{e+\Delta t}z^{\prime }{}_{e+\Delta t}$. The coordinate of the target in relative to the new space coordinate system is $S_{t+\Delta t}^{\prime }\left(x_{t+\Delta t}^{\prime },y_{t+\Delta t}^{\prime },z_{t+\Delta t}^{\prime }\right)$, and the image plane coordinate is $A_{t+\Delta t}^{\prime }\left(a_{x,t+\Delta t}^{\prime },a_{y,t+\Delta t}^{\prime }\right)$. Assuming that before every rotation in this process, the camera coordinate system is the space coordinate system, its mathematical model can be described as shown in Equation (66):

(66) $$O^{\prime }{}_{e+\Delta t}=R_{e+\Delta t}*O_{e}$$

where $O_{e}$ is the set space coordinate at moment t, i.e., the standard coordinate system; $O^{\prime }{}_{e+\Delta t}$ is the camera coordinate after rotation at moment Δt, equivalent to the transition coordinate system introduced for calculating the rotation. In $R_{e+\Delta t}=R_{\alpha }\times R_{\beta }$, $R_{e+\Delta t}$ is the rotation matrix. Its coordinate transformation, i.e., the process of movement, is as shown in Figure 19. The target object moves from Point A to Point B, and yaw angle α and pitch angle β are adjusted through PTZ to ensure that the visual axis is aligned with the target object and achieve target tracking.

The 2-DOF PTZ angle is adjusted to ensure the coincidence of optical center and rotation center; the tracking motion of the visual axis can be broken into the rotation motion around x axis and y axis in the camera coordinate system. The matrix is shown in Equation (67):

(67) $$R_{e+\Delta t}=\left[\begin{array}{ccc} \cos\Delta \beta & 0 & -\sin\Delta \beta \\ -\sin\Delta \beta \sin\Delta \alpha & \cos\Delta \alpha & -\sin\Delta \alpha \cos\Delta \beta \\ -\sin\Delta \beta \cos\Delta \alpha & \sin\Delta \alpha & \cos\Delta \alpha \cos\Delta \beta \end{array}\right]$$

The workflow is as follows: in coordinate system o, the starting position of the UAV is the origin of coordinates, the starting position of target is $A(x_{t},y_{t},z_{t})$ and the detection angle of airborne camera is 120°. After a short time Δt, the target position is $A(x_{t+\Delta t},y_{t+\Delta t},z_{t+\Delta t})$. (1) The Jetson Nano collects a video stream through the UAV front-end camera. (2) When the target is found, it marks the target and sends the target frame coordinates and color images to the target tracking algorithm for initialization. (3) The color image is sent to the target tracking algorithm for iterative updating, and the next color image is re-executed in the third step. (4) According to the color image and the corresponding target frame coordinate information, the UAV flight height, speed, and attitude angle are controlled in order to enable the UAV to approach the target. The Jetson Nano detects distance via the front-end ultrasonic ranging module to keep a safe distance from the target. (5) After entering the range, the Arduino control board executes the fire control program, drives the two-degrees-of-freedom steering gear pan tilt, quickly corrects angles α and β between the collimation and the target, automatically executes the shooting command after aiming, drives the gel ball blaster unit motor to launch through the relay, and completes its task of hitting the target.

In ideal conditions, the new coordinate should be the same as the image plane coordinate at moment *t*. In the actual movement process, due to the extraction error of image plane coordinates in the target recognition process and the tracking error of the control system, an because of the continuous movement of the target during the tracking process, the visual feedback system suffers an upper limit of tracking speed. In order to further improve the speed and accuracy of target tracking, higher requirements are put forward for the design of a PTZ controller.

#### 3.3.2. Anti-Windup PID Control Algorithm

In the design process of the pan tilt control system, the structural strength and response speed of the steering gear are limited; in general, this is referred to as plant input limitation. In addition, the PTZ module requires frequent switches to different modes, such as from follow mode to target attack mode, which is known as plant input substitution. Due to the existence of input limitation and displacement, the input and output of the control system are sometimes unequal, which leads to further variation in the closed-loop response of the control system, resulting in the windup phenomenon. The PID controller is widely used across various aspects of control system design. In order to eliminate static error, the windup phenomenon is inevitable in the integral part of the controller. The fast-tracking task results in higher requirements in the design of the PTZ control system. Typically, the control system takes a small signal as input in the process of debugging and operation. When the PTZ of the steering gear quickly follows and adjusts the firing angle, the control signal input is given a large range of sudden change, which is prone to large overshoots and vibrations, affecting the stability of the entire flight control system.

In view of the windup phenomenon in the PTZ control system, we have established the PTZ model of the steering gear, analyzed the influence of the structure, speed and force of the steering gear on tracking and proposed an Anti-Windup PID controller to reduce the influence of actuator saturation and improve the dynamic response performance of the steering gear PTZ. Firstly, we ignore the nonlinear effect of saturation caused by the actuator limitation of the steering gear and take the deviation between the expected position of the aim point and the actual position as the input value, integrate the saturation error, and weaken the saturation effect by adjusting the adaptive coefficient. When the pan tilt of the steering gear is adjusted slightly, the compensator does not work. When the PTZ of the steering gear is adjusted rapidly and at a large angle, the path and time information and large signal are taken as input values. The PID controller with anti-integral saturation compensation will play a role in ensuring the control performance as the system is saturated. The input video stream resolution is 1080 × 720 pixels. The center abscissa (540,360) of the frame image pixel of the video stream is taken as the given value, and the center abscissa of the target frame is taken as the output value and negative feedback, all of which form a closed-loop control loop. After the difference between the given value and the feedback value is passed through the Anti-Windup PID controller, it is sent to the Arduino control board through the serial port. From this, the angular speed of rotation is calculated in order to control the rotation of the pan tilt of the steering gear so that the target is in the center of the image.

**Figure 20:**
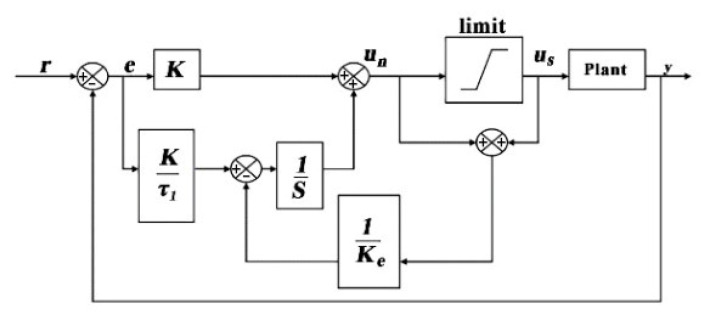
Back-calculation anti-windup Proportional Integral Derivative (PID) structure.

In this simulation experiment, the Anti-Windup PID controller adopts back-calculation, and the structure is shown in Figure 20.

Anti-Windup PID controller output expression:

(68) $$u_{n}=\frac{KK_{e}\left(\tau_{1}s+1\right)}{\tau_{1}\left(K_{e}s+1\right)}e+\frac{1}{K_{e}s+1}u_{s}$$

In the determination of distance from the target, we adopted the ultrasonic ranging scheme. The threshold value is set to 10 m. When the ultrasonic device detects that it is 10 m from the target, the UAV stops moving toward it. The 10 m distance can effectively ensure the safety of the UAV, and at the same time, remain within effective expelling range. In this paper, the binocular camera is not used for video streaming and target depth information collection, because the data volume of the binocular camera is too large, and the maximum processing speed of the airborne processor Jetson Nano can only reach three frames per second. The real-time performance of the program is poor.

## 4. Experimental Results

**Figure 21:**
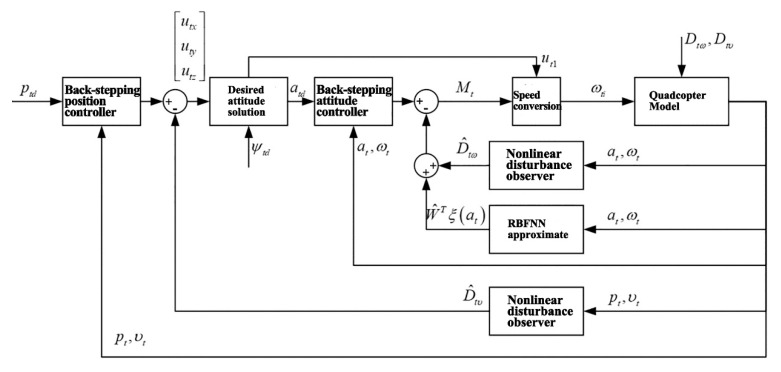
Structural diagram of quadcopter trajectory tracking controller.

The experiment is divided into five stages:

In the first stage, in Section 2.2 and Section 3.1, the back-stepping method was applied to design the attitude controller and trajectory tracking controller of quadcopters, and the Lyapunov stability of the said controllers was demonstrated. An experimental simulation based on the quadcopter model was conducted to validate the correctness of the designed controllers. To demonstrate the advanced nature of the controllers, the traditional PID controller was used as a comparable counterpart. The diagram of the trajectory tracking controller designed for the quadcopter is shown in Figure 21.

Under the Matlab2018/Simulink environment, the trajectory tracking of the designed controller was simulated, where a fixed step size of 0.001 s was adopted. The specified trajectory was given by Equation (69), and the initial values for the attitude angle, its angular velocity, position, and speed were all zero in the initial state. The following parameters for the controller were selected, as shown in Table 4.

(69) $$\left\{ \begin{array}{l} x_{d}=0,\;y_{d}=0,\;z_{d}=-0.2t;\;t\in (0,5]\\ x_{d}=0.5\times \left(t-5\right),\;y_{d}=0,\;z_{d}=-1;\;t\in (5,10]\\ x_{d}=2.5,\;y_{d}=0.3\times \left(t-10\right),\;z_{d}=-1;\;t\in (10,15]\\ x_{d}=2.5-0.5\times \left(t-15\right),\;y_{d}=1.5,\;z_{d}=-1;\;t\in (15,20]\\ x_{d}=0,\;y_{d}=1.5-0.3\times \left(t-20\right),\;z_{d}=-1;\;t\in (20,25]\\ x_{d}=0,\;y_{d}=0,\;z=-1+0.2\times \left(t-25\right);\;t\in (25,30]\;\\ \psi_{d}=0\;rad,\;t\in (0,5]\\ \psi_{d}=0.25\;rad,\;t\in (5,30] \end{array}\right.$$

The external disturbance terms $D_{t\upsilon }\left(t\right)$ and $D_{t\omega }\left(t\right)$ are shown by Equation (70) and Equation (71), respectively.

(70) $$d_{a}\left(t\right)=\left[\begin{array}{c} -0.6785\cos\left(1.2t+30^{{}^{\circ}}\right)\\ -0.8422\sin\left(1.5t+50^{{}^{\circ}}\right)\\ -0.5114\cos\left(1.3t+20^{{}^{\circ}}\right) \end{array}\right]$$

(71) $$\left[\begin{array}{c} d_{tu}\left(t\right)\\ d_{tv}\left(t\right)\\ d_{tw}\left(t\right) \end{array}\right]=\left[\begin{array}{c} -1.3187\cos\left(0.7t+40^{{}^{\circ}}\right)\\ -1.3562\sin\left(0.5t+20^{{}^{\circ}}\right)\\ -2.5716\cos\left(0.8t+50^{{}^{\circ}}\right) \end{array}\right]$$

**Figure 22:**
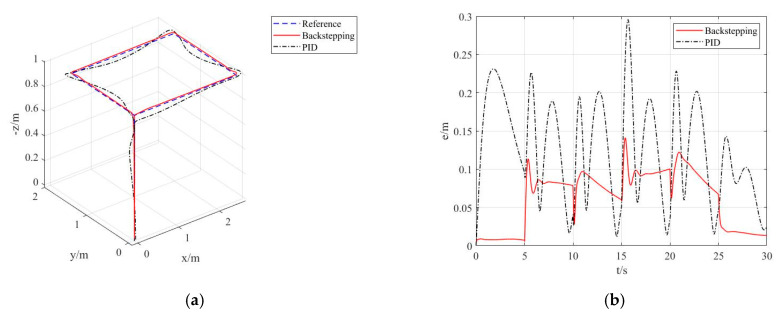

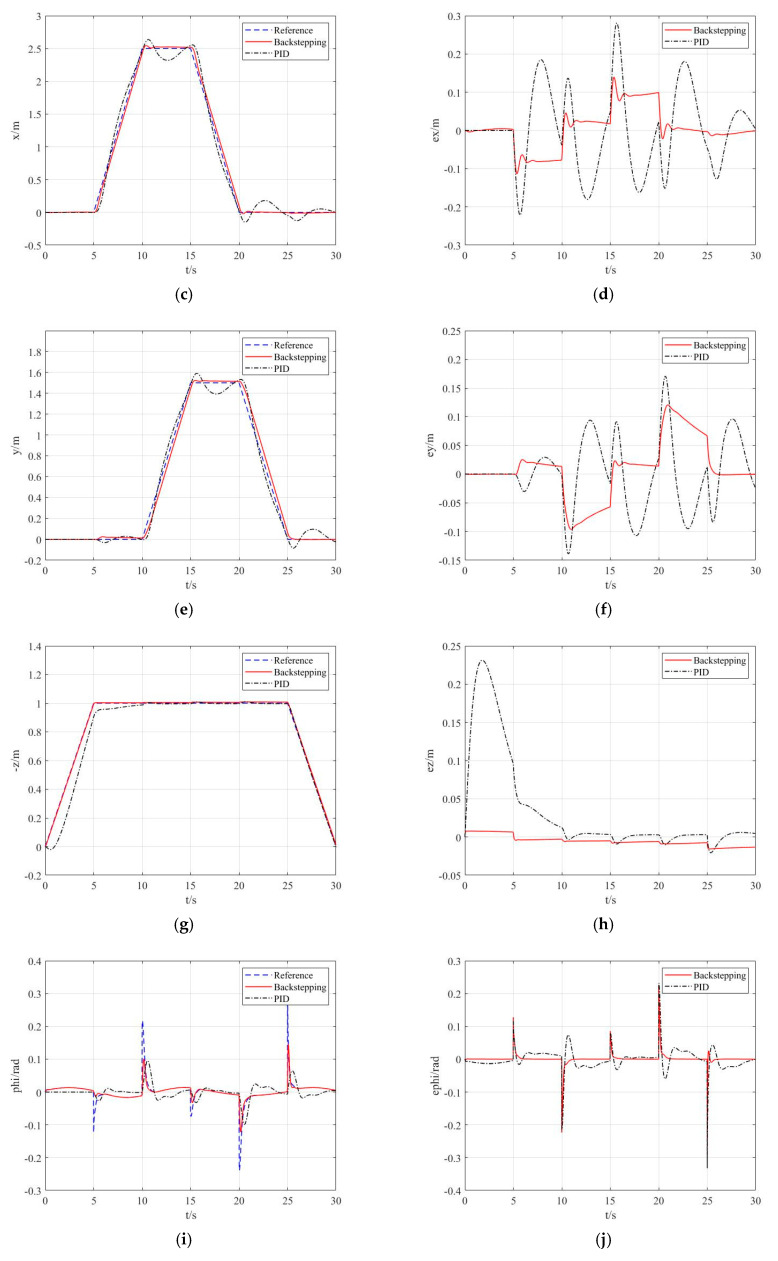

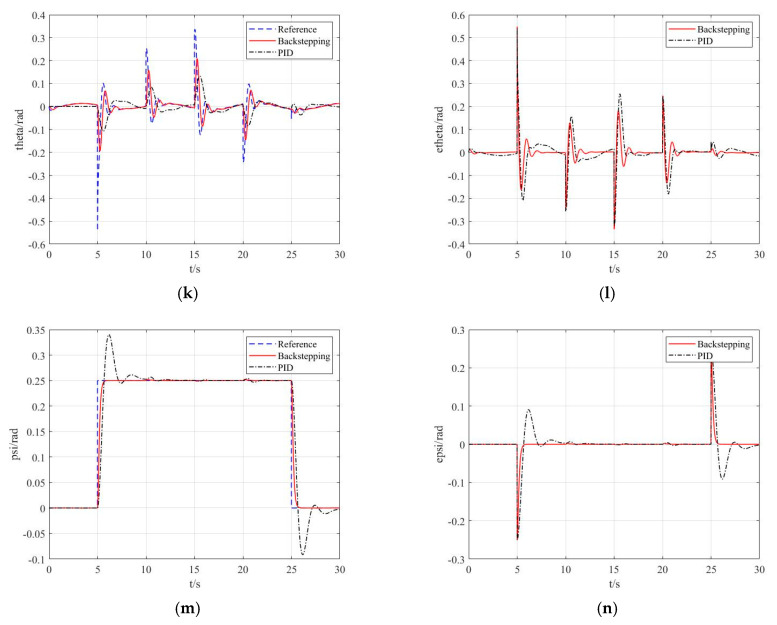
Curve of quadcopter trajectory tracking responses. (**a**) 3D curve of trajectory tracking. (**b**) Curve of trajectory tracking errors. (**c**) Curve of trajectory tracking along the *x*-axis. (**d**) Curve of trajectory tracking errors along the *x*-axis. (**e**) Curve of trajectory tracking along the *y*-axis. (**f**) Curve of trajectory tracking errors along the *y*-axis. (**g**) Curve of trajectory tracking along the *z*-axis. (**h**) Curve of trajectory tracking errors along the *z*-axis. (**i**) Curve of roll angle responses. (**j**) Curve of roll angle tracking errors. (**k**) Curve of pitch angle responses. (**l**) Curve of pitch angle tracking errors. (**m**) Curve of yaw angle responses. (**n**) Curve of yaw angle tracking errors.

The simulation results are shown in Figure 22.

As can be seen from the simulation results in Figure 22, the quadcopter has fairly good performance in terms of tracking the desired trajectories under the designed trajectory tracking controller. As can be seen from Figure 22b, when tracking 3D trajectories, the maximum error is kept within 0.15 m; comparatively, the maximum error under the PID controller reaches 0.3 m. Furthermore, the average error of the designed controller is also lower than that of the PID controller. As seen in Figure 22c–f, the maximum tracking errors along the *x*- and *y*-axis are around 0.1 m, and both the maximum and average errors are lower than those of the PID controller. As seen in Figure 22g–h, the tracking error remains within 0.02 m along the *z*-axis—an evidently better performance than the PID controller.

As seen in Figure 22i,n, both the designed attitude controller and the PID attitude controller can effectively maintain the quadcopter’s attitude. However, the attitude controller designed in this paper can enable the three attitude angles of the quadcopter to rapidly converge towards the desired angles.

In the meantime, as seen in Equation (69), the desired trajectories of the quadcopter when t∈(0,5]s and t∈(25,30]s are straight upwards and straight downwards. Under the effect of external disturbances, the tracking errors along the *x*- and *y*-axis remain within 0.01 m, and around 0.02 m along the *z*-axis. These lay the foundation for quadrotor flight stability. During outdoor testing with disturbance factors like varied wind force, wind direction, and mass, the controller also delivered fairly good stability and reliability.

**Figure 23:**
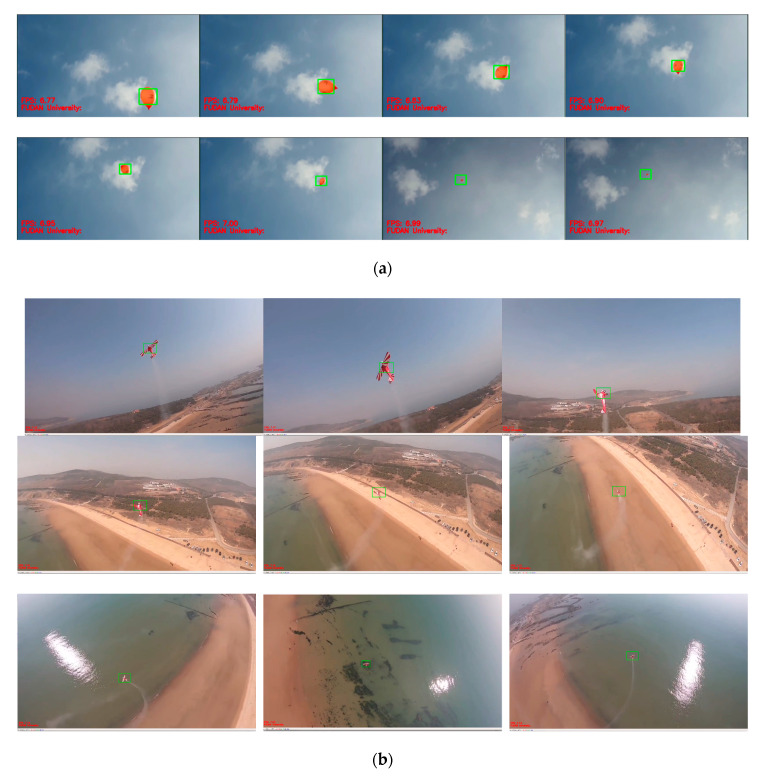
Target tracking test: (**a**) balloon in the air; (**b**) fixed-wing model aircraft in flight.

In the second stage, we ran a test for target tracking algorithm. The test was conducted with the selected balloon in the air and the fixed-wing model aircraft in flight. The result showed that fast and accurate tracking can be realized in airborne image processing with a velocity of around 7 FPS when the size, speed, posture, and background of the target change. As shown in Figure 23. Table 5 listed the tracking rate of different types of targets.

**Figure 24:**
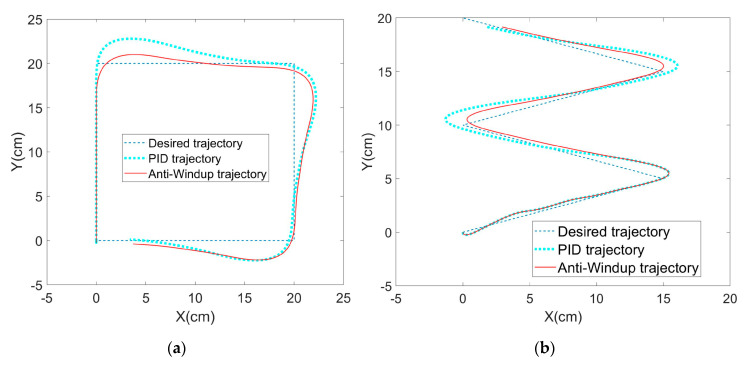
Simulation test of PTZ controller trajectory tracking: (**a**) shape of square; (**b**) shape of Z.

In the third stage, we ran a trajectory tracking MATLAB simulation experiment of the steering gear PTZ. Expected trajectories in shapes of square and Z were set up to test the performance of Anti-Windup PID controller. Compared with a traditional PID controller, the results showed that, under the large signal input with rapid changes, the overshoot of the Anti-Windup PID controller is smaller with faster shrink, and its PTZ controlling performance is better than that of the traditional PID controller, as shown in Figure 24.

**Figure 25:**
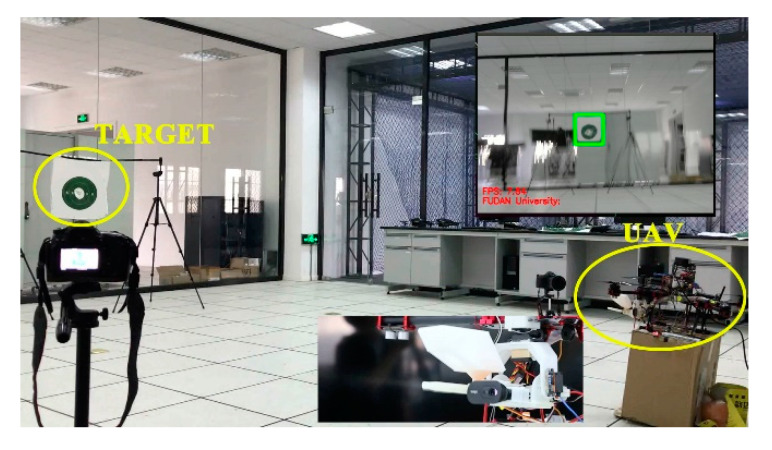
Indoor static test.

In the fourth stage, we ran an indoor static test. The UAV was placed on a box with a height of 0.5 m. The target was set 1.5 m high and 8 m away from the UAV. When the target was calibrated, Jetson nano, Arduino, and the steering gear PTZ located the target within 2 s, corrected the shooting trajectory, and triggered the shooting procedure. The gel ball trajectory was scattered within the range of 10 cm. High-speed cameras had been used to shoot the target, and then the number of impact points in each target area was counted through slow-motion playback to calculate the accuracy of PTZ launch. Hit rate of indoor static test (deviation <20 cm) was 94.44%. An expected result was achieved in the experiment. Indoor accuracy test, as shown in Table 6 and Figure 25.

**Figure 26:**
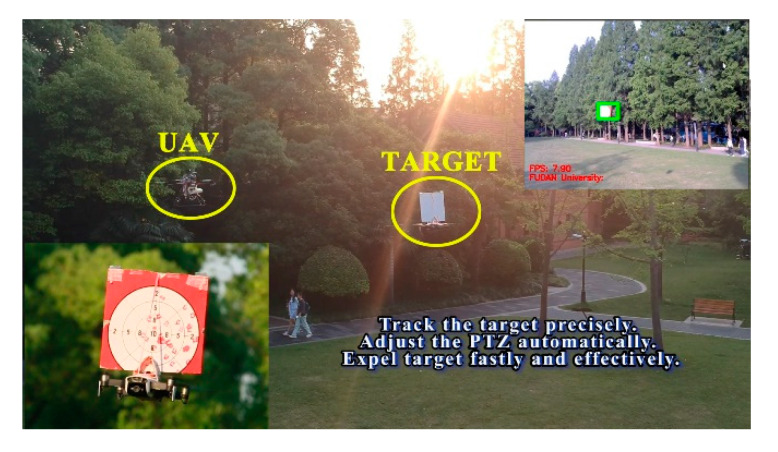
Outdoor dynamic expelling test.

**Figure 27:**
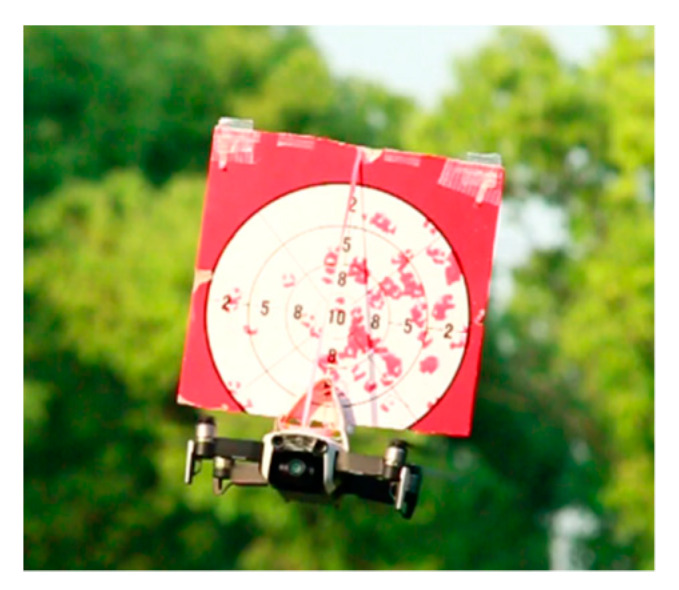
Target image.

**Figure 28:**
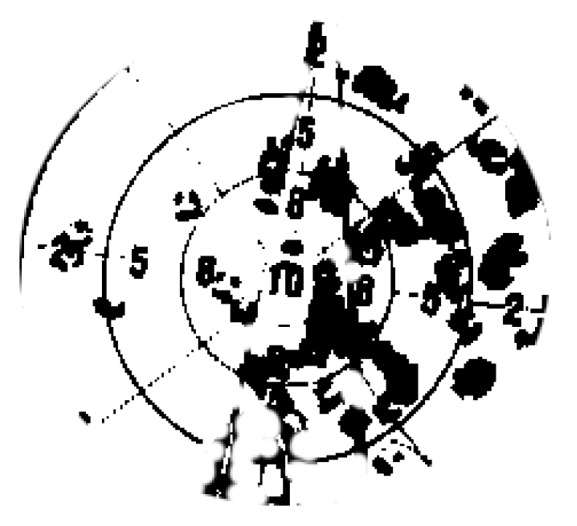
Target in grayscale.

In the fifth stage, we launched an outdoor dynamic expelling test. As shown in Table 7 and Figure 26. The target was fixed on the DJI (DJ-Innovations, Shenzhen, China) UAV, which could move randomly in the air. The UAV we designed could follow the flight with a response speed of only 0.5 s. Meanwhile, it processed the target tracking program at high speed (the rate in testing environment was 7.2 Fps), and executed the trajectory correcting program (10 times/s) and the launching program (15 times/s). The gel ball dispersion was within 20 cm in field tests. Figure 27 is a close-up shot of the target, and Figure 28 is the gray-scale image of the target, which shows the distribution of impact points visually. The test method is the same as above. Hit rate of outdoor dynamic test (deviation <20 cm) was 82.98%. In the outdoor dynamic shooting test, fast and effective expulsion of the target UAV was achieved.

## 5. Conclusions and Future Work

We have designed an integrated identification, tracking, and expelling quadcopter UAV system based on computer vision, proposed a real-time image recognition, location, and tracking plan based on the YOLO and KCF algorithms, and designed corresponding target tracking and expelling strategies. We have made a prototype UAV, conducted target recognition and tracking tests, a PTZ control algorithm test, an indoor static test, and outdoor dynamic expelling testing, and assessed the design plan in different scenarios. According to the experiment, an automatic target identification and tracking system is designed based on the YOLO and KCF algorithms, and the ability to identify and track the target with high speed in a complex environment is realized. Additionally, the high-speed steering gear PTZ that is designed by the Anti-Windup algorithm is adopted to effectively ensure the ability of operating the aiming and expelling device with high speed and precision. The UAV system based on computer vision introduced in this paper can identify, track, and expel small targets and changeable targets quickly in complex environments, thus realizing the air target control with high efficiency and low cost.

We have demonstrated that the computer vision technology has great feasibility and significance in the design of an integrated identification, tracking and expelling UAV, with broad application prospects in civil fields. However, some problems still deserve further research:

1. Meteorological conditions may have a great influence on target recognition, especially the light intensity, dust, smoke, and other interference factors.
2. Based on the multiple-target and tracking studies of the YOLO and KCF algorithms, an estimation of the moving speed and trajectory of multiple targets, and the prediction of the hazard level.
3. The impact of vibration on PTZ and attack precision of targets in the operating process of the trigger mechanism. The electrically controlled simulated launch device used in this experiment has a small recoil force, so the impact of vibration is not considered for the time being. In future practical applications, the vibrations created by the target expulsion device cannot be ignored, and a damping device should be designed.
4. Unlike high-cost schemes, this paper focuses on a low-cost target-expelling scheme, using gel balls as combat ammunition. In a real flight environment, wind power, wind direction, and ammunition weight are the factors that must be considered, and an analysis of hitting points spread across different ranges is needed.

The design of the obstacle avoidance system based on multisensor information fusion has been completed and successfully tested. It will be introduced in detail in future articles. A video (Supplementary Materials) link to the whole experiment has been included at the end of this article and gives part of the obstacle avoidance test. You are welcome to click on it and check our results.

## Figures and Tables

**Table 1:** Comparison of monitoring technologies.

Monitoring Technologies	Drone Signature	Localization/Tracking Method	Detection Range	Can it Expel Targets?	Challenges
Radar	Micro-Doppler	Doppler-based tracking delay-based localization	≤3000 m	NO	Low radar cross-section, low speed and altitude
Audio	Time-frequency feature	DOA-based localization	40–300 m	NO	High ambient noise
Video	Appearance feature motion feature	Motion-based tracking	100–1000 m	NO	Occlusion indistinguishable small object
RF	Communication channel	Received-signal-strength (RSS)/DOA-based localization	≤1000 m	YES	EMI

**Table 2:** Quadcopter parameters.

Parameters	Values	Units
m	1.94	kg
g	9.81	m/s^2^
$l_{t}$	265	mm
$J_{tr}$	$4.92\times 10^{-6}$	kg·m^2^
$J_{xx}$	$2.37\times 10^{-3}$	kg·m^2^
$J_{yy}$	$3.51\times 10^{-3}$	kg·m^2^
$J_{zz}$	$5.31\times 10^{-3}$	kg·m^2^

**Table 3:** You Only Look Once (YOLO) real-time recognition location results.

Parameters	a	b	c	d
Bounding box x coordinate	0.5028	0.2920	0.7434	0.5055
Bounding box y coordinate	0.4783	0.4867	0.4675	0.6494
Bounding box length	0.5752	0.5758	0.4379	0.5634
Bounding box height	0.7651	0.7626	0.7602	0.6964
Actual x coordinate (cm)	0	–30	30	0
Actual y coordinate (cm)	0	0	0	10
Actual altitude (cm)	100	100	100	100
Recognition x coordinate (cm)	0.0354	−26.30	31.21	0.0707
Recognition y coordinate (cm)	–0.1591	–0.2299	–0.2387	10.96
Recognition altitude (cm)	98.10	98.16	133.2	102.8

**Table 4:** Simulation parameters.

Back-Stepping Controller	Nonlinear Disturbance Observer	PID Controller
$\Lambda_{ta}=diag\left\{5,5,5\right\}$	$L_{t\upsilon }=diag\left\{40,40,40\right\}$	$K_{xp}=5$, $K_{xi}=5$, $K_{xd}=5$
$\Lambda_{t\omega }=diag\left\{10,10,10\right\}$	$L_{t\omega }=diag\left\{50,50,50\right\}$	$K_{yp}=5$, $K_{yi}=5$, $K_{yd}=5$
$\Lambda_{tp}=diag\left\{1,1,0.5\right\}$		$K_{zp}=10$, $K_{zi}=3$, $K_{zd}=10$
$\Lambda_{t\upsilon }=diag\left\{5,5,10\right\}$		$K_{\phi p}=5$, $K_{\phi i}=3$, $K_{\phi d}=4$
		$K_{\theta p}=5$, $K_{\theta i}=3$, $K_{\theta d}=4$
		$K_{\psi p}=5$, $K_{\psi i}=2$, $K_{\psi d}=1.5$

**Table 5:** Tracking rates of different types of targets.

Target Type	Balloon	Fixed-Wing Model	DJI Phantom4	DJI Mavic Pro	DJI Mavic Air
**Frame rate (Fps)**	6.83	7.17	6.52	6.14	6.10

**Table 6:** Indoor accuracy test.

Distance from Target Center (cm)	0–5	6–10	11–15	16–20	20+
**Number of impact points**	32	28	17	8	5

**Table 7:** Outdoor accuracy test.

Distance from Target Center (cm)	0–5	6–10	11–15	16–20	20+
**Number of impact points**	12	19	26	21	16

## Supplementary Materials

Video of the whole experiment: https://youtu.be/IPUHHmi-Gto.

## Abbreviation

UAV	Unmanned Aerial Vehicle
DOF	Degrees of Freedom
PTZ	Pan/Tilt/Zoom
YOLO	You Only Look Once
KCF	Kernel Correlation Filter
PID	Proportional Integral Derivative
RF	Radio Frequency
FMCW	Frequency Modulated Continuous Wave
MIMO	Multiple Input Multiple Output
DOA	Direction of Arrival
RSS	Received-Signal-Strength
HOG	Histogram of Oriented Gradient
3D	Three-Dimensional
EMI	Electromagnetic Interference
RBFNN	Radial Basis Function Neural Network
DNN	Deep Neural Network
CNN	Convolutional Neural Network
R-CNN	Regions with Convolutional Neural Network Features
FPS	Frames Per Second
CSRT	Channel and Spatial Reliability Tracking
MIL	Multiple Instance Learning
MOSSE	Minimum Output Sum of Squared Error
TLD	Tracking Learning Detection
HOG	Histogram of Oriented Gradient
